# Representability of algebraic topology for biomolecules in machine learning based scoring and virtual screening

**DOI:** 10.1371/journal.pcbi.1005929

**Published:** 2018-01-08

**Authors:** Zixuan Cang, Lin Mu, Guo-Wei Wei

**Affiliations:** 1 Department of Mathematics, Michigan State University, East Lansing, Michigan, United States of America; 2 Computer Science and Mathematics Division, Oak Ridge National Laboratory, Oak Ridge, Tennessee, United States of America; 3 Department of Biochemistry and Molecular Biology, Michigan State University, East Lansing, Michigan, United States of America; 4 Department of Electrical and Computer Engineering, Michigan State University, East Lansing, Michigan, United States of America; University of Illinois at Urbana-Champaign, UNITED STATES

## Abstract

This work introduces a number of algebraic topology approaches, including multi-component persistent homology, multi-level persistent homology, and electrostatic persistence for the representation, characterization, and description of small molecules and biomolecular complexes. In contrast to the conventional persistent homology, multi-component persistent homology retains critical chemical and biological information during the topological simplification of biomolecular geometric complexity. Multi-level persistent homology enables a tailored topological description of inter- and/or intra-molecular interactions of interest. Electrostatic persistence incorporates partial charge information into topological invariants. These topological methods are paired with Wasserstein distance to characterize similarities between molecules and are further integrated with a variety of machine learning algorithms, including k-nearest neighbors, ensemble of trees, and deep convolutional neural networks, to manifest their descriptive and predictive powers for protein-ligand binding analysis and virtual screening of small molecules. Extensive numerical experiments involving 4,414 protein-ligand complexes from the PDBBind database and 128,374 ligand-target and decoy-target pairs in the DUD database are performed to test respectively the scoring power and the discriminatory power of the proposed topological learning strategies. It is demonstrated that the present topological learning outperforms other existing methods in protein-ligand binding affinity prediction and ligand-decoy discrimination.

## Author summary

Conventional persistent homology neglects chemical and biological information during the topological abstraction and thus has limited representational power for complex chemical and biological systems. In terms of methodological development, we introduce advanced persistent homology approaches for the characterization of small molecular structures which can capture subtle structural difference. We also introduce electrostatic persistent homology to embed physics in topological invariants. These approaches encipher physics, chemistry and biology, such as hydrogen bonds, electrostatics, van der Waals interactions, hydrophobicity and hydrophilicity, into topological fingerprints which, although cannot literally recast into physical interpretations, are ideally suitable for machine learning, particularly deep learning, rendering topological learning algorithms. In terms of applications, we construct a structure-based virtual screening model which outperforms other existing methods. This competitive model on the DUD database is derived by assessing the performance of a comprehensive collection of topological approaches proposed in this work and introduced in our earlier work, on the PDBBind database. The topological features constructed in this work can readily be applied to other biomolecular problems where the characterization of proteins or small molecules is needed.

This is a *PLOS Computational Biology* Methods paper.

## Introduction

Arguably, machine learning has become one of the most important developments in data science and artificial intelligence. With its ability to extract features of various levels hierarchically, deep convolutional neural networks (CNNs) have made breakthroughs in image processing, video, audio, and computer vision [1, 2], whereas recurrent neural networks have found success in analyzing sequential data, such as text and speech [3–6]. Deep learning algorithms are able to automatically extract high-level features and discover intricate patterns in large data sets. In general, one of the major advantages of machine learning algorithms is their ability to deal with large and diverse data sets and uncover complicated relationships.

Recently, machine learning has become an indispensable tool in biomolecular data analysis and structural bioinformatics. Almost every computational problem in molecular biophysics and biology, such as the predictions of solvation free energy, solubility, partition coefficient, protein-ligand binding affinities, mutation induced protein stability change, molecular multipolar electrostatics, virtual screening, etc., has machine learning based approaches that are either parallel or complementary to their physics based counterparts. The success of deep learning has fueled the rapid growth in several areas of biological science [3, 5, 6], including bioactivity of small-molecule drugs [7–10] and genetics [11, 12], where large data sets are available.

A key component of a learning machine based on biomolecular structures is featurization, that is translating the 3D structures of biomolecules to features. While the degrees of freedom of the original biomolecular structures are large and vary among different molecules, it is almost inevitable that information loss happens with dimension reduction during featurization. Besides the choice of learning models, the performance of a predictor heavily depends on how the features are extracted. Although deep learning has been known to be powerful for the automatic extraction of features from original inputs such as images, deep learning based models directly taking biomolecules as inputs are not as competitive as the state-of-art machine learning models with carefully designed features, due to the intrinsic complexity of biomolecules [13].

Biomolecules can be characterized by geometric features, electrostatic features, high-level (residue and global level) features, and amino-acid sequence features based on physical, chemical, and biological understandings [14]. Geometric features, such as coordinates, distances, angles, surface areas [15–17] and curvatures [18–21], are important descriptors of biomolecules [22–24]. However, geometric features often involve too much structural detail and are frequently computationally intractable for large biomolecular data sets. Electrostatic features include atomic partial charges, Coulomb potentials, atomic electrostatic solvation energies, and polarizable multipolar electrostatics [25]. These descriptors become essential for highly charged biomolecular systems, such as nucleic acid polymers and some protein-ligand complexes. High-level features refer to pKa values of ionizable groups and neighborhood amino acid compositions, such as the involvement of hydrophobic, polar, positively charged, negatively charged, and special case residues. Sequence features consist of secondary structures, position-specific scoring matrix (PSSM), and co-evolution information. Sequence features and annotations provide a rich resource for bioinformatics analysis of biomolecular systems. Topology offers a new unconventional representation of biomolecules. Topology can describe biomolecules in a variety of ways [26]. Some of the most powerful topological features are obtained from multi-component persistent homology or element specific persistent homology (ESPH) [14, 27]. Recently, we carried out a comprehensive comparison of the performance of geometric features, electrostatic features, high-level features, sequence features and topological features, for the prediction of mutation induced protein folding free energy changes of four mutation data sets [14]. Surprisingly, topological features outperform all the other features [14].

Unlike geometry, topology is well known for its power of simplification to geometric complexity [28–35]. The global description generated by classical topology is based on the concept of neighborhood and connectedness. If a space can be continuously deformed to another, they are considered to have the same topological features. In this sense, topology can not distinguish between a folded protein and its unfolded form if only covalent bonds are considered. Such property prevents the use of classical topology for the characterization of biomolecular structures. Instead of using topology to describe a single configuration of connectivity, persistent homology scans over a sequence of configurations induced by a filtration parameter and renders a sequence of topological invariants, which partially captures part of geometric features. Persistent homology has been applied to biomolecular systems in our earlier works [26].

In mathematics, persistent homology is a relatively new branch of algebraic topology [29, 36]. When dealing with proteins and small molecules, it is conventional to consider atoms as point clouds. For a given point cloud data set, one type of persistent homology turns each point into a sphere with their radii systematically increasing. The corresponding topological invariants and their persistence over the varying radius values can be computed. Therefore, this method embeds multiscale geometric information in topological invariants to achieve an interplay between geometry and topology. Consequently, persistent homology captures topological structures continuously over a range of spatial scales. It is called persistent homology because at each given radius, topological invariants, i.e., Betti numbers, are practically calculated by means of homology groups. In the past decade, much theoretical formulation [37–46] and many computational algorithms [47–52] have been developed. One-dimensional (1D) topological invariants generated from persistent homology is often visualized by persistence barcodes [53, 54] and persistence diagrams [55]. In recent years, multidimensional persistence has attracted much attention [43, 56] in hope that it can better characterize the data shape when there are multiple measurements of interest.

Persistent homology has been applied to various fields, including image/signal analysis [57–62], chaotic dynamics verification [63, 64], sensor networks [65], complex networks [66, 67], data analysis [68–72], shape recognition [73–75], and computational biology [76–79]. Compared with traditional computational topology [80–82] and/or computational homology, persistent homology inherently adds an additional dimension, i.e., the filtration parameter. The filtration parameter can be used to embed important geometric or quantitative information into topological invariants. As such, the importance of retaining geometric information in topological analysis has been recognized [83], and persistent homology has been advocated as a new approach for handling big and high dimensional data sets [54, 68, 84–86]. Recently, we have introduced persistent homology for mathematical modeling and/or prediction of nano-particles, protein unfolding, and other aspects of biomolecules [26, 87]. We proposed the molecular topological fingerprint (TF) to reveal *topology-function relationships* in protein folding and protein flexibility [26]. We established some of the first quantitative topological analyses in our persistent homology based predictions of the curvature energy of fullerene isomers [87, 88]. We have also shown correlation between persistence barcodes and energies computed with physical models during molecular dynamics experiments [26]. Moreover, we have introduced the first differential geometry based persistent homology that utilizes partial differential equations (PDEs) in filtration [88]. Most recently, we have developed a topological representation to address additional measurements of interest, by stacking the persistent homology outputs from a sequence of frames in molecular dynamics or a sequence of different resolutions [89, 90]. We have also introduced one of the first uses of topological fingerprints for resolving ill-posed inverse problems in cryo-EM structure determination [91]. In 2015, we constructed one of the first integrations of topology and machine-learning and applied it to protein classification involving tens of thousands of proteins and hundreds of tasks [92]. We also developed persistent-homology based software for the automatic detection of protein cavities and binding pockets [93].

Despite much success, it was found that persistent homology has a limited characterization power for proteins and protein complexes, when applied directly to biomolecules [92]. Essentially, biomolecules are not only complex in their geometric constitution, but also intricate in biological constitution. In fact, the biological constitution is essential to biomolecular structure and function. Persistent homology that is designed to reduce the geometric complexity of a biomolecule neglects biological information. To overcome this difficulty, we have introduced multi-component persistent homology or element specific persistent homology (ESPH) to recognize the chemical constitution during the topological simplification of biomolecular geometric complexity [14, 27, 94]. In ESPH, the atoms of a specific set of element types in a biomolecule are selected so that specific chemical information, such as hydrophobicity or hydrophilicity, is emphasized in each selection. Our ESPH is not only able to outperform other geometric and electrostatic representations in large and diverse data sets, but is also able to shed light on the molecular mechanism of protein-ligand binding, such as the relative importance of hydrogen bond, hydrophilicity and hydrophobicity at various spatial ranges [27].

The objective of the present work is to further explore the representability and reduction power of multi-component persistent homology for biomolecules and small molecules. To this end, we take a combinatorial approach to scan a variety of element combinations and examine the characterization power of these components. Additionally, we also propose a multi-level persistence to study the topological properties of non-covalent bond interactions. This approach enables us to devise persistent homology to describe the interactions of interest between atoms that are connected by weak non-covalent bonds and delivers richer representation especially for small molecules. Moreover, realizing that electrostatics are of paramount importance in biomolecules and to enhance the power of our topological representation, we introduce electrostatic persistence, which embeds charge information in topological invariants, as a new class of features in multi-component persistent homology. The aforementioned approaches can be realized via the modification of the distance matrix with a more abstract setting, for example, Vietoris-Rips complex. The complexity reduction is guaranteed in the 1D topological representation of 3D biomolecular structures. Obviously, the multi-component persistent homology representation of biomolecule leads to a higher machine learning dimensionality compared to the original single component persistent homology for a biomolecule. Therefore, it is subject to overfitting or overlearning problem in machine learning theory. Fortunately, gradient boosting trees (GBT) method is relatively insensitive to redundant high dimensional topological features [14]. Finally, since the components can be arranged as a new dimension ordered by their feature importance, multi-component persistent homology barcodes are naturally a two-dimensional (2D) representation of biomolecules. Such a 2D representation can be easily used as image-like input data in a deep CNN architecture, with different topological dimensions, i.e., 0, 1, and 2, being treated as channels. Such a topological deep learning approach addresses the nonlinear interactions among important element combinations while keeping the information from less important ones. Barcode space metrics, such as bottleneck distance and more generally, Wasserstein distance [95, 96], offer a direct description of similarity between molecules and can be readily used with nearest neighbor regression or kernel based methods. The performance of Wasserstein distance for protein-ligand binding affinity predictions is examined in this work.

After assessing the new method’s ability to represent small molecules and protein-compound complexes, the derived model is used for virtual screening. Virtual screening computationally screens a collection of small molecules to identify those who can potentially bind to the protein target. There are mainly two types of virtual screening which are ligand-based and structure-based. Ligand-based approaches depend on a measurement of similarity among small molecules using either 2D or 3D structural information of small molecules. Structure-based approaches attempt to dock the small molecule candidate to the protein target and determine if the candidate is a potential ligand based on the top docking poses. The performance of structure-based virtual screening methods heavily depends on the quality of the docking method and the accuracy of the post-docking scoring method. Our effort focuses on the development of a topology based method for the latter part. It has been shown that using machine learning or deep learning based methods to rescore the docking poses can significantly boost the performance [97, 98]. For the models such as ensemble of trees and classical neural networks, carefully constructed features are needed. For example, a neural network based method NNScore uses a collection of derived features such as the count of hydrogen bonds and electrostatics of close contacts to describe the protein-compound complex [97]. Another class of deep learning based methods feed lower level features to deep neural networks and relies on the neural networks to automatically extract higher-level features. For example, DeepVS first computes features on each atom involved in the docking interface and feed this information to a deep neural network starting with convolution layers to hierarchically extract higher-level features [98].

The rest of this manuscript is organized as follows. *Section Methods* is devoted to introducing methods and algorithms. We present multi-component persistent homology, multi-level interactive persistent homology, vectorized persistent homology representation and electrostatic persistence. These formulations are crucial for the representability of persistent homology for biomolecules. Machine learning algorithms associated with the present topological data analysis are briefly discussed. Results are presented in *Section Results*. We first consider the characterization of small molecules. More precisely, the cross-validation of protein-ligand binding affinities prediction via solely ligand topological fingerprints is studied. We illustrate the excellent representability of our multi-component persistent homology by a comparison with a method using physics based descriptors. Additionally, we investigate the representational power of the proposed topological method on a few benchmark protein-ligand binding affinity data sets, namely, PDBBind v2007, PDBBind v2013, PDBBind v2015 and PDBBind v2016 [99]. These data sets contain thousands of protein-ligand complexes and have been extensively studied in the literature. Results indicate that multi-component persistent homology offers one of most powerful representations of protein-ligand binding systems. The aforementioned study of the characterization of small molecules and protein-ligand complexes leads to an optimal selection of features and models to be used for virtual screening. Finally, we consider the directory of useful decoys (DUD) database to examine the representability of our multi-component persistent homology for virtual screening to distinguish actives from non-actives. The DUD data set used in this work has a total of 128,374 ligand-target and decoy-target pairs containing 3961 active ligand-target pairs, and involves 40 protein targets from six families. A large number of state-of-the-art virtual screening methods have been applied to this data set. We demonstrate that the present multi-component persistent homology outperforms other methods with reported results on this benchmark. This paper ends with a conclusion.

## Results

Rational drug design and discovery have rapidly evolved into some of the most important and exciting research fields in medicine and biology. These approaches potentially have a profound impact on human health. The ultimate goal is to determine and predict whether a given drug candidate will bind to a target so as to activate or inhibit its function, which results in a therapeutic benefit to the patient. Virtual screening is an important process in rational drug design and discovery which aims to identify actives of a given target from a library of small molecules. There are mainly two types of screening techniques, ligand-based and structure-based. Ligand-based approaches depend on the similarity among small molecule candidates. Structure-based approaches try to dock a candidate molecule to the target protein and judge the candidate with the modeled binding affinity based on docking poses. Various molecular docking software packages have been developed for these purposes. Molecular docking involves both pose generation and binding affinity scoring. Currently, pose generation is quite robust while scoring power is still limited. Therefore, knowledge-based rescoring methods using machine learning or deep learning approaches can improve scoring accuracy [97, 98, 100]. We also apply our topological learning method as a rescoring machine to rerank the candidates based on docking poses generated by docking software.

**Fig 1:**
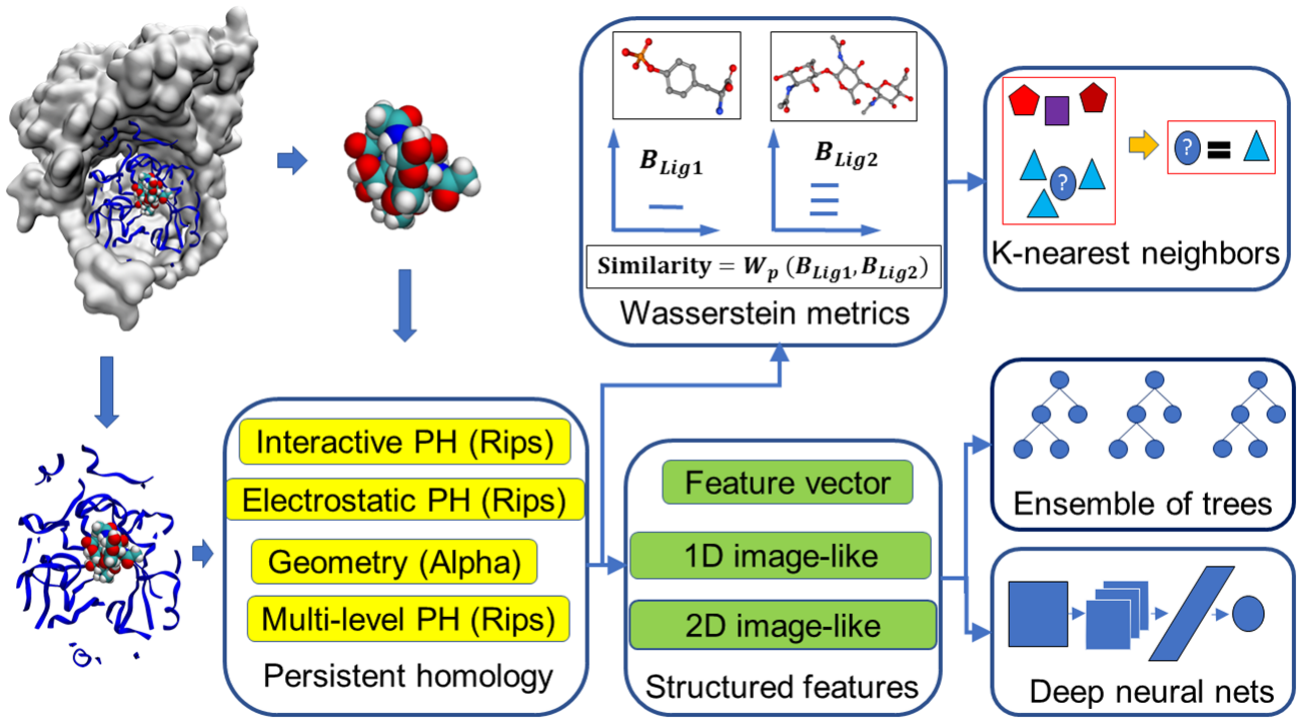
An illustration of the topology based machine learning algorithms used in scoring and virtual screening.

This section explores the representational power of the proposed persistent homology methods for the prediction of protein-ligand binding affinities and the discrimination of actives and non-actives for protein targets. To this end, we use the present method to investigate three types of problems. First, we develop topological learning models for ligand based protein-ligand binding affinity predictions. This problem is designed to examine the representability of the proposed topological methods for small molecules. Then, we develop topological learning models for protein-ligand complex based binding affinity prediction. This problem enables us to understand the capability of the proposed topological learning methods for dealing with protein-ligand complexes. Finally, we examine the structure-based classification of active ligands and decoys which are highly possible to be non-actives, i.e., structure-based virtual screening (VS). The optimal selection of features and methods are determined by studying the first two applications and this finding leads to the main application studied in this work, the topological structure-based virtual screening. Computational algorithms used in this study are illustrated in Fig 1.

### Ligand based protein-ligand binding affinity prediction

In this section, we address the representation of small molecules by element specific persistent homology, especially the proposed multi-level persistent homology designed for small molecules.

#### Data set

To assess the representational ability of the present persistent homology algorithms on small molecules, we use a high quality data set of 1322 protein-ligand complexes with binding affinity data involving 7 protein clusters introduced earlier (denoted as S1322) [101]. It is a subset of the PDBBind v2015 refined set and its detail is given in the Supplementary material 1 of Ref. [101]. We consider a ligand based approach to predict the binding affinities of protein-ligand complexes in various protein clusters. As such, only the ligand information is used in our topological analysis. The ligand structures are taken from PDBBind database without modification. Numbers of ligands in protein clusters range from 94 to 333.

#### Models and performance

Two models, i.e., TopBP-KNN(Ligand) and TopBP-ML(Ligand), are constructed. TopBP-KNN(Ligand) is used to directly assess the representation power of persistent homology for small molecules and TopBP-ML(Ligand) is the final practical model. The results are shown in Table 1. All the gradient boosting trees models take the setup described in *Section Methods/Machine learning algorithms/Gradient boosting trees*.

**Table 1:** Pearson correlation coefficients (RMSE in kcal/mol) of ligand based topological model on the S1322 dataset.

Methods	CL 1 (333)	CL 2 (264)	CL 3 (219)	CL 4 (156)	CL 5 (134)	CL 6 (122)	CL 7 (94)	Average
TopBP-KNN(Ligand)	0.698(1.66)	0.817(1.28)	0.620(1.68)	0.645(1.41)	0.756(1.68)	0.658(1.68)	0.739(1.31)	0.705(1.49)
TopBP-ML(Ligand) (5-fold)	0.713(1.60)	0.843(1.15)	0.693(1.51)	0.670(1.35)	0.831(1.34)	0.698(1.56)	0.737(1.26)	0.741(1.40)
FFT-BP (5-fold) [101]	(1.93)	(1.32)	(2.01)	(1.61)	(2.02)	(2.06)	(1.71)	(1.81)

The numbers in the first row show the number of entries in each protein cluster. The performance is reported as Pearson correlation coefficient (root mean squared error in kcal/mol). The median performance of 20 random 5-fold cross validation results is reported for TopBP-ML(Ligand). The results reported for TopBP-KNN(Ligand) are obtained by leave-one-out validation within each protein cluster with *k* = 3 for the KNN model.

In TopBP-ML(Ligand), we process the geometry, the shape, and the covalent bond information of the small molecules using alpha complex, and the non-covalent intramolecular interactions using multi-level persistent homology with Rips complex. The features used are A-B012-E-S-GBT and R-B012-M1-S-GBT as described in *Section Discussion/Ligand based protein-ligand binding affinity prediction*. Gradient boosting trees method is used.

In TopBP-KNN(Ligand), we represent the small molecules with a collection of barcodes from element specific persistent homology calculations. Wasserstein distance with *p* = 2 is applied to measure similarities between two barcodes. The similarity between each pair of small molecules is then measured by taking the average of the Wasserstein distances between all considered barcodes. K-nearest-neighbor (KNN) regression is then applied to the measured similarity. In detail, the 6 barcodes considered are, R-B0-E-KNN, R-B1-E-KNN, R-B2-E-KNN, R-B0-M1-KNN, R-B1-M1-KNN, and R-B2-M1-KNN as described in *Section Discussion/Ligand based protein-ligand binding affinity prediction*. Leave-one-out validation within each protein cluster with *k* = 3 is used for this model.

In Table 1, FFT-BP 5-fold cross validation results were obtained based on multiple additive regression trees and a set of physical descriptors, including geometry, charge, electrostatic interactions, and van der Waals interactions for S1322 set [101]. Since multiple additive regression trees is also an implementation of the GBT used in the present work, it is appropriate to compare the FFT-BP results with the GBT results in this work to assess representation power of topological features. It is interesting to note that judging by RMSE, both sets of current topological descriptors have more predictive power than the physical descriptors built on protein-ligand complexes constructed in our earlier work [101]. These physical descriptors were constructed from sophisticated surface areas, molecular volumes, van der Waals interactions, charges computed by quantum mechanics, and Poisson-Boltzmann theory based electrostatics [101]. The success of topological descriptors implies the existence of an alternative and potentially more powerful description of the complex biomolecular world.

### Complex based protein-ligand binding affinity prediction

In this section, we develop topological representations of protein-ligand complexes.

#### Data sets

The PDBBind database provides a comprehensive collection of structures of protein-ligand complexes and their binding affinity data [99, 102]. The original experimental data in Protein Data Bank (PDB) [103] are selected to PDBBind database based on certain quality requirements and are curated for applications. As shown in Table 2, this database is expanding on a yearly basis. It has become a common resource for benchmarking computational methods and algorithms for protein-ligand binding analysis and drug design. Popular data sets include version 2007 (v2007), v2013, and v2015. Among them, v2013 core set and v2015 core set are identical. A large number of scoring functions has been tested on these data sets. The latest version, v2016, has an enlarged core set, which contains 290 protein-ligand complexes from 58 protein families. Therefore, this test set should be relatively easier than v2015 core set, whose 195 complexes involve 65 protein families. The core sets are constructed by choosing 3 samples with median, maximum, and minimum binding affinity from each protein family for v2007, v2013, and v2015 sets. The core set for v2016 was constructed similarly but with 5 samples from each protein family.

**Table 2:** Description of the PDBBind datasets.

Version	Refined set	Training set	Core set (test set)	Protein families
v2007	1300	1105	195	65
v2013	2959	2764	195	65
v2015	3706	3511	195	65
v2016	4057	3767	290	58

Number of complexes or number of protein families in PDBBind data sets used in the present binding affinity prediction. Here training sets are set to the corresponding refined sets, excluding the complexes in the corresponding test sets (i.e., core sets). Protein families refer to those in the corresponding core sets.

#### Model and performance

Two models TopBP-ML(Complex) and TopBP-DL(Complex) are introduced. The results are shown in Table 3. All the gradient boosting trees models take the setup described in *Section Methods/Machine learning algorithms/Gradient boosting trees*.

**Table 3:** Pearson correlation coefficients (RMSE in kcal/mol) of different protein-ligand complex based approaches on PDBBind datasets.

**Core set predictions**					
Methods	v2007	v2013	v2015	v2016	Average
TopBP(Complex)	0.827 (1.93)	0.808 (1.95)	0.812 (1.92)	0.861 (1.65)	0.827 (1.86)
TopBP-ML(Complex)	0.818 (2.01)	0.804 (2.00)	0.797 (1.99)	0.848 (1.74)	0.817 (1.94)
TopBP-DL(Complex)	0.806 (1.95)	0.781 (1.98)	0.799 (1.91)	0.848 (1.64)	0.809 (1.87)
RF::VinaElem^a^	0.803 (1.94) [104]	0.752 (2.03) [105]	-	-	-
RI-Score [106] ^b^	0.803 (1.99)^c^	-	0.762 (2.05)^c^	0.815 (1.85)	-
**Refined set 5-fold cross validations**					
Methods	v2007	v2013	v2015	v2016	Average
TopBP-ML(Complex)	0.752 (1.95)	0.768 (1.75)	0.781 (1.71)	0.785 (1.71)	0.771 (1.78)
RI-Score [106] ^d^	-	-	-	0.747 (1.83)	-

Pearson correlation coefficients with RMSE (kcal/mol) in parentheses for predictions by different methods are listed. For the tests on core sets, the models are trained with the corresponding refined set minus the core set. Five-fold cross validation is done on refined sets. Results of TopBP-ML(Complex) are the medians of 50 repeated runs. For TopBP-DL(Complex), 100 independent models are generated at first. A consensus model is built by randomly choosing 50 models out of the 100, and this process is repeated 1000 times with the median reported. TopBP(Complex) is a consensus model combining TopBP-ML(Complex) and TopBP-DL(Complex). Each time, 50 single deep learning models are randomly selected to form TopBP-DL(Complex) and a TopBP-ML(Complex) model is randomly selected. The average of the two is taken as the output for TopBP(Complex). This process is repeated 1000 times with the median reported.

*^a^* The authors did not specify the number of repeated experiments and whether the reported performance is the best or the median of the experiments.

*^b^* The medians of Pearson correlation coefficient among the repeated experiments are listed.

*^c^* Only the best RMSEs among the repeated experiments are reported.

*^d^* The median results are reported.

In TopBP-ML(Complex), alpha complex is used to describe the arrangement of carbon and heavy atom networks, while Rips complex with different distance matrices is used to describe the protein-ligand interactions from the perspective of interaction distances and strength of electrostatics interactions. In detail, the features used are R-B0-I-C, R-B0-CI-S, A-B12-E-S as described in *Section Discussion/Complex based protein-ligand binding affinity prediction*, and those used in TopBP-ML(Ligand).

With the idea that a sequence of element combinations ordered by their importance in gradient boosting trees models can make an extra dimension of the description, we build a 2D convolutional neural network with one spatial dimension and one dimension of element combination. We combine this 2D CNN with a 1D CNN with the pairwise interaction inputs. For the construction of 2D input, the reader is referred to *Section Feature generation from topological invariants*. The 1D image-like inputs consist of two parts both generated by the counts in bins method described in *Section Feature generation from topological invariants*. For the 0th dimensional barcodes from interactive persistent homology of the 36 pairs of atom types ({C,N,O,S} from protein and {C,N,O,S,P,F,Cl,Br,I} from ligand), the interval [0, 50] Å is divided into equal length subintervals of length 0.25 Å. For the 0th dimensional barcodes from interactive persistent homology for electrostatics of the 50 pairs of atom types ({C,N,O,S,H} from protein and {C,N,O,S,P,F,Cl,Br,I,H} from ligand), the parameter interval of [0, 1] is divided into equal length subintervals of length 0.01. These two 1D image-like features have sizes 200 × 36 and 100 × 50. The network architecture is given in *Section Methods/Machine learning algorithms/Deep convolutional neural networks*.

The final model TopBP(Complex) takes the average of TopBP-ML(Complex) and TopBP-DL(Complex) with the assumption that the errors made by the two approaches are only partially correlated and thus averaging over them may cancel part of the errors. As a result, TopBP(Complex) delivers the best prediction performance on all four testing sets.

### Structure-based virtual screening

In this section, we examine the performance of the proposed method for the main application in this paper, which is structure-based virtual screening which involves protein-compound complexes obtained by attempting to dock the candidates to the target proteins. The dataset is much larger than the two applications on protein-ligand binding affinity prediction which makes parameter tuning very time consuming. Therefore, the best performing procedures in ligand-based binding affinity prediction and protein-ligand-complex-based binding affinity prediction are applied in this virtual screening application.

#### DUD data set

The directory of useful decoys (DUD) [107, 108] is used to benchmark our topological approach for virtual screening. The DUD data set contains 40 protein targets from six classes, i.e., nuclear hormone receptors, kinases, serine proteases, metalloenzymes, folate enzymes, and other enzymes. A total of 3,961 active ligand-target pairs were identified from literature. The number of ligands for each target ranges from tens to hundreds. At most 36 decoys were constructed for each ligand, from the ZINC database of commercially available compounds [109]. At the first step, the ZINC database of 3.5 million compounds was reduced to a database of 1.5 million compounds with similarity less than 0.9 to the ligands. The similarity was measured by Tanimoto coefficient on CACTVS type 2 fingerprints. The decoys were selected so that they possess similar physical properties to the ligands but have dissimilar molecular topology (topology in the sense of chemistry, not mathematical topology). A total of 32 physical properties were used including molecular weight, partition coefficient, and number of hydrogen bonding groups. This results in a total of 128,374 compound-target pairs. A discrepancy between calculated partial charges for the ligand and decoy sets was reported for the original release 2 of DUD datasets, which makes it trivial for virtual screening methods to distinguish between the two categories using those charges [110]. In this work, we use the data with recalculated Gasteiger charges for both ligand and decoy sets given by Armstrong *et al.* [110] in AutoDock Vina and our electrostatic persistence.

#### Data processing

In structure-based virtual screening, the possible complex structures of the target protein and the small molecule candidate are required. For the DUD dataset, the structures of the 40 protein targets, the ligands, and the decoys are given, and we generate the protein-compound complexes by using docking software. To this end, we first add missing atoms to the proteins by using the profix utility in Jackal software package [111]. The receptors and ligands or decoys are prepared using the scripts prepare_receptor4.py and prepare_ligand4.py provided by the AutoDockTools module in MGLTools package (version 1.5.6) [112]. The bounding box of the binding site is defined as a cube with edge size equal to 27 Å, centered at the geometric center of the crystal ligand. AutoDock Vina (version 1.1.2) [113] is used to dock the ligands or decoys to the receptors. The option exhaustiveness is set to 16 and all the other parameters are set to their default values. In each docking experiment, the pose having the lowest binding free energy reported by AutoDock Vina, is used by the reranking models.

#### Evaluation

Two measurements, the enrichment factor (EF) and the area under the receiver operating characteristic curve (AUC), are used to evaluate each method’s ability of discriminating actives from decoys. The AUC is defined as

$$\begin{array}{c} \text{AUC}=1-\frac{1}{N_{a}}\sum_{i=1}^{N_{a}}\frac{N_{d}^{i}}{N_{d}}, \end{array}$$ (1)

where *N*_*a*_ is the number of active ligands, *N*_*d*_ is the total number of decoys, and $N_{d}^{i}$ is the number of decoys that are higher ranked than the *i*th ligand [98]. An AUC value of 0.5 is the expected value of a random selection, whereas a perfect prediction results in an AUC of 1. The EF at *x*% denoted by EF_*x*%_ evaluates the quality of the set of top *x*% ranked compounds, by comparing the percentage of actives in the top *x*% ranked compounds to the percentage of actives in the entire compound set. It is defined as

$$\begin{array}{c} {\text{EF}}_{x\%}=\frac{N_{a}^{x\%}}{N^{x\%}}\cdot \frac{N}{N_{a}}, \end{array}$$ (2)

where $N_{a}^{x\%}$ is the number of active ligands in the top *x*% ranked compounds, *N*^*x*%^ is the number of top *x*% ranked compounds, *N* is the total number of compounds, and *N*_*a*_ is the total number of active ligands.

To evaluate the performance of various methods on the DUD data set, the entries associated with one protein target are used as the test set in the experiment on this protein target [98]. For the selection of the training set of a given protein target, we follow a procedure given in the literature [107], where the entries associated to the rest of the proteins, excluding those that are within the same class of the testing protein and those that have reported positive cross-enrichment with the testing protein, are taken as the training set. The 40 proteins are split into 6 classes [100]. A detailed list of proteins that are excluded from the training set of each protein is given in Table F in S1 Text.

#### Topology based machine learning models

Our topology based machine learning model, called *TopVS-ML*, relies on manually constructed features and utilizes ensemble of trees methods. For the complex with the small molecules (i.e., ligands and decoys) docked to the receptor, features R-B0-I-BP, R-B0-CI-S, and A-B12-E-S are used (see *Section Discussion/Complex based protein-ligand binding affinity prediction*), whereas features R-B012-M1-S and A-B012-E-S (see *Section Discussion/Ligand based protein-ligand binding affinity prediction*) are used for the small molecules. The gradient boosting trees method, random forest method, and extra trees method are employed as voters. The averaged probabilities output by the three methods are used for the classifier to decide the class of the testing samples. The modules *GradientBoostingClassifier*, *RandomForestClassifier*, and *ExtraTreesClassifier* in the scikit-learn package [114] (version 0.17.1) are used. The parameters for the three modules are listed in Table 4. TopVS-ML achieves a performance of AUC = 0.83, EF_2%_ = 8.6, EF_20%_ = 3.4. These values are the median values of 10 repeated experiments. Table G in S1 Text lists the result of each single experiment confirming that the performance is consistent across each repeated run.

**Table 4:** Parameters used in machine learning.

Method	Parameters
GBT	n = 2000, s = 0.5, cw = 100:1, lr = 0.01, mf = sqrt
RF	n = 2000, cw = balanced_subsample
ET	n = 2000, cw = balanced_subsample

The parameters used for the ensemble of trees methods while the other parameters are set to default. GBT: gradient boosting trees. RF: random forest. ET: extra trees. n: n_estimators. s: subsample. cw: class_weight. lr: learning_rate. mf: max_feature.

#### Topology based deep learning model

Our topology based deep learning model, called *TopVS-DL*, relies on 1D image-like inputs for protein-compound complexes and manually constructed features for the compounds. The 2D representation used in binding affinity problem is not used here due to the intractable data size. The manually constructed features for the compounds are R-B012-M1-S and A-B012-E-S as described in *Section Discussion/Ligand based protein-ligand binding affinity prediction*. The 1D image-like inputs consisted of three parts are all generated by the counts in bins method described in *Section Feature generation from topological invariants*. (1) For the 0th dimensional barcodes from interactive persistent homology of the 36 pairs of atom types ({C, N, O, S} from protein and {C, N, O, S, P, F, Cl, Br, I} from ligand), the interval [0, 25] Å is divided into equal length subintervals of length 0.25 Å. The barcodes used here are identical to the barcodes in feature R-B0-I-BP. This results in a 1D image-like feature with size 100 × 36. (2) For the 0th dimensional barcodes from interactive persistent homology for electrostatics of the 50 pairs of atom types ({C, N, O, S, H} from protein and {C, N, O, S, P, F, Cl, Br, I, H} from ligand), the parameter interval of [0, 1] is divided into equal length subintervals of length 0.01. The barcodes used are identical to the barcodes in feature R-B0-CI-S. This results in a 1D image-like feature with size 100 × 50. (3) Alpha complex based persistent homology is applied to all carbon atoms and all heavy atoms. The computation is done on the complex as well as only the protein with a cutoff distance of 12 Å from the ligands. The interval [0, 12] Å is divided into equal length subintervals of length 0.125 Å. Counts in bins method is applied to the 0th, 1st, and 2nd dimensional barcodes. The features are generated for persistent homology computation of the complex and the protein. The features for the complex and the difference between the features for complex and protein are finally used. This results in a 1D image-like feature of size 96 × 32. The detailed network architecture is listed in *Section Methods/Machine learning algorithms/Deep convolutional neural networks*. A consensus model is constructed by taking the average over 25 single models trained independently. TopVS-DL achieves a performance of AUC = 0.81, EF_2%_ = 9.1, EF_20%_ = 3.2.

#### The final model

Same as the idea of taking the average output of different ensemble of trees models as the final output in TopVS-ML, we add TopVS-DL as another voter to TopVS-ML to construct a final model, called *TopVS*. Such consensus approach takes the average over different models with the hope that different models make partially uncorrelated errors which are possible to cancel out when averaged. The performance on each of 40 protein targets is reported in Table 5. We have also generated virtual screening results of AutoDock Vina (ADV) based on the computed binding free energy by ADV and compared them with those of the present TopVS in terms of enrichment factors and the areas under the receiver operating characteristic curve (AUC). A comparison of average AUC with those from a large number of methods is given in Table 6.

**Table 5:** Performance on each protein in DUD dataset.

Target	ADV			TopVS		
	EF_2%_	EF_20%_	AUC	EF_2%_	EF_20%_	AUC
ACE	4.1	1.4	0.42	5.1	3.1	**0.81**
AChE	4.7	2.8	**0.67**	1.4	1.9	0.65
ADA	0.0	0.4	0.49	7.8	4.5	**0.90**
ALR2	2.0	2.7	**0.74**	4.9	1.5	0.68
AmpC	2.4	0.2	0.34	0.0	1.0	**0.58**
AR	17.0	3.8	0.81	20.1	4.2	**0.90**
CDK2	9.0	2.4	0.64	7.6	4.1	**0.88**
COMT	13.1	1.4	0.56	17.4	2.9	**0.73**
COX1	9.9	2.8	0.76	11.8	3.6	**0.86**
COX2	20.7	3.9	0.86	23.3	4.9	**0.97**
DHFR	6.4	2.8	0.82	12.6	4.7	**0.96**
EGFr	3.4	1.6	0.63	16.4	4.8	**0.95**
ER_agonist_	17.8	3.3	**0.84**	10.0	2.8	0.81
ER_antagonist_	10.2	2.3	0.70	1.3	2.8	**0.83**
FGFr1	0.4	0.8	0.44	15.1	4.8	**0.95**
FXa	1.0	1.3	0.63	2.1	4.4	**0.89**
GART	0.0	1.9	**0.75**	2.6	0.7	0.48
GPB	0.0	0.9	0.48	1.4	1.5	**0.66**
GR	5.7	1.2	0.57	1.3	3.4	**0.84**
HIVPR	5.6	2.6	0.74	8.9	4.4	**0.91**
HIVRT	8.2	1.9	0.64	11.7	4.0	**0.88**
HMGR	0.0	0.9	0.53	14.4	5.0	**0.96**
HSP90	0.0	0.9	0.64	9.6	4.5	**0.93**
InhA	13.4	1.9	0.56	22.7	4.5	**0.95**
MR	16.7	4.0	0.82	0.0	4.3	**0.87**
NA	0.0	0.3	0.37	1.5	3.8	**0.87**
P38 MAP	1.4	1.7	0.59	18.4	4.5	**0.94**
PARP	4.2	2.7	0.71	0.0	1.7	0.71
PDE5	8.0	1.9	0.61	6.9	3.4	**0.86**
PDGFrb	3.5	0.5	0.32	26.5	4.9	**0.97**
PNP	0.0	0.7	0.59	7.9	4.3	**0.89**
PPARg	17.7	3.4	**0.82**	0.6	1.8	0.72
PR	1.9	1.1	0.52	9.4	4.1	**0.91**
RXRa	28.2	4.8	**0.95**	12.8	3.2	0.83
SAHH	10.4	3.0	0.80	4.5	3.9	**0.84**
SRC	5.6	2.3	0.71	24.6	4.9	**0.98**
thrombin	8.3	2.6	0.72	4.1	2.4	**0.79**
TK	0.0	0.9	0.56	6.9	2.5	**0.65**
trypsin	3.1	1.9	0.58	0.0	2.0	**0.78**
VEGFr2	10.2	2.2	0.63	24.9	4.7	**0.96**
Average	6.9	2.0	0.64	9.5	3.5	**0.84**

The median results of 10 repeated runs with different random seeds (for the TopVS-ML part) are reported. The best AUC in each row is marked in bold. The left block of AutoDock Vina (ADV) results are acquired from the ADV runs with the binding free energy reported by ADV.

**Table 6:** AUC comparison of different methods on DUD dataset.

Method	AUC	Ref.
TopVS	0.84	
DeepVS-ADV	0.81	[98]
ICM^a^	0.79	[115]
NNScore1-ADV^b^	0.78	[97]
Glide SP^a^	0.77	[116]
DDFA-ALL	0.77	[100]
DDFA-RL	0.76	[100]
NNScore2-ADV^b^	0.76	[97]
DDFA-ADV	0.75	[100]
DeepVS-Dock	0.74	[98]
DDFA-AD4	0.74	[100]
Glide HTVS^b^	0.73	[97]
Surflex^a^	0.72	[116]
Glide HTVS	0.72	[116]
ICM	0.71	[115]
RAW-ALL	0.70	[100]
AutoDock Vina^b^	0.70	[97]
Surflex	0.66	[116]
Rosetta Ligand	0.65	[100]
AutoDock Vina	0.64	[100]
ICM	0.63	[116]
FlexX	0.61	[116]
Autodock4.2	0.60	[100]
PhDOCK	0.59	[116]
Dock4.0	0.55	[116]

^*a*^Tuned by expert knowledge.

^*b*^Determined using a different data set of decoys.

## Discussion

### Ligand based protein-ligand binding affinity prediction

**Fig 2:**
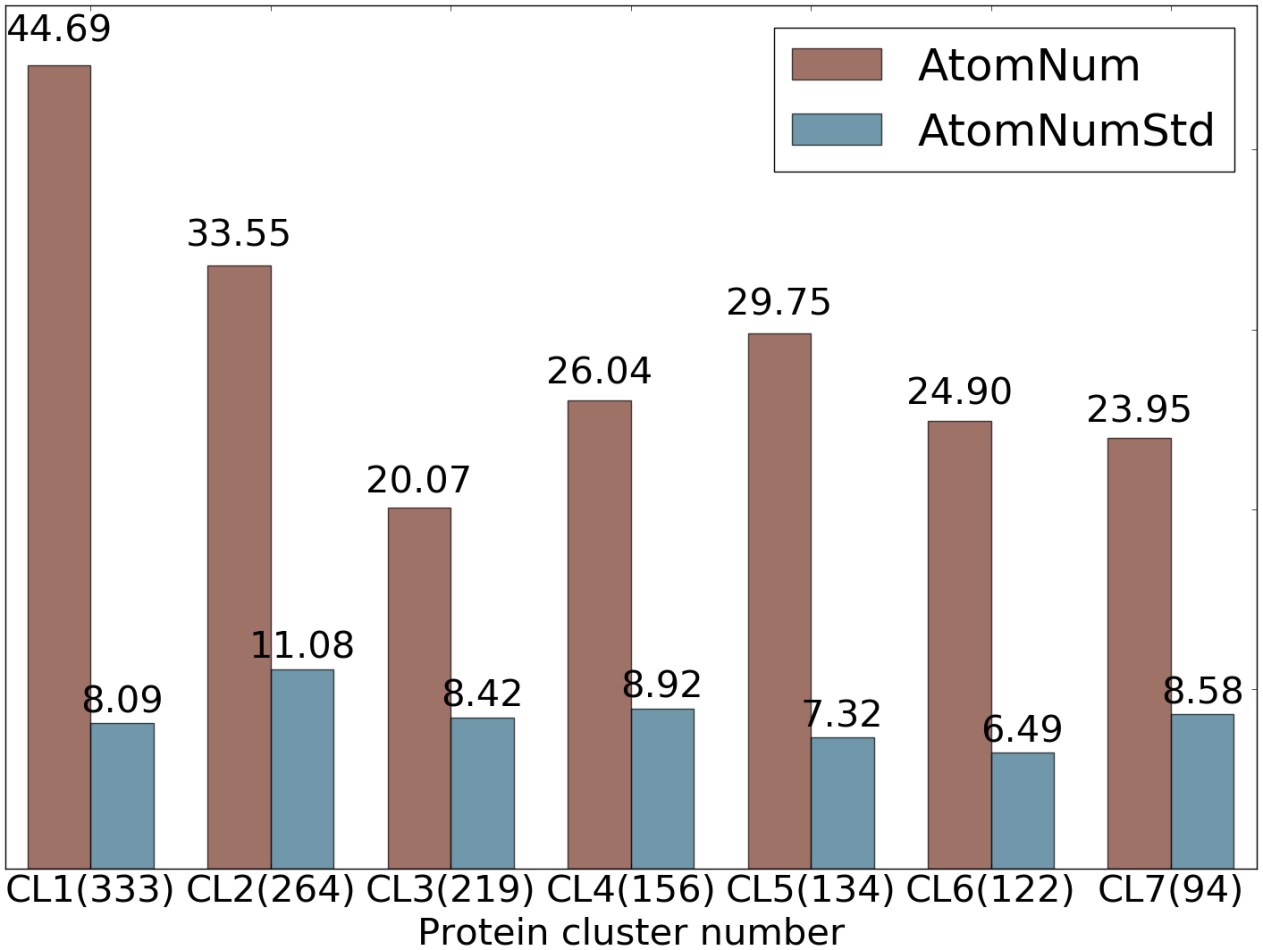
Statistics of ligands in 7 protein clusters in S1322 dataset. The average numbers of heavy atoms of a ligand in each protein cluster are shown in red and the standard deviations of number of heavy atoms across each protein cluster are shown in blue. The number of ligands in each cluster is given in parentheses.

We conduct several experiments on ligand based protein-ligand binding affinity prediction in this section which leads to the final models. To examine the strength and weakness of different sets of features and models, we first show a statistics fact of the S1322 data set of 7 protein clusters in Fig 2. The details of the S1322 data set is given in *Section Results/Ligand based protein-ligand binding affinity prediction*. All the gradient boosting trees models take the setup described in *Section Methods/Machine learning algorithms/Gradient boosting trees*.

#### Feature vectors for gradient boosting trees

In this test, Rips complex based and alpha complex based persistent homology computations up to 2nd dimension are performed for a variety of atom collections with different element types using the Euclidean metric and multi-level distance defined in Eq (3). Two types of features are generated and are denoted by *F*^*C*^, which is a combination of $F_{b}^{C}$, $F_{d}^{C}$, and $F_{p}^{C}$, and *F*^*S*^, which is a combination of $F_{b}^{S}$, $F_{d}^{S}$, and $F_{p}^{S}$. The construction of features *F*^*C*^ and *F*^*S*^ are described in *Section Feature generation from topological invariants*. For sets of the 0th dimensional bars, only $F_{d}^{C}$ and $F_{d}^{S}$ are computed. In each protein cluster, 10-fold or 5-fold cross validation is repeated 20 times for each subset of feature vectors depending on selected element type. The median Pearson correlation coefficients and the root-mean-square error (RMSE) in kcal/mol are reported. For Rips complex, both level 0 computation with distance matrix **M** and level 1 computation with distance matrix ${\tilde{M}}^{1}$ as defined in Eq (4) are performed. A comparison of these results is shown in S1 Text Table B. The results corresponding to alpha complex are shown in S1 Text Table A. The average performance for alpha complex and Rips complex has a Pearson correlation coefficient of 0.987.

#### Barcode space metrics for k-nearest neighbor regression

**Fig 3:**
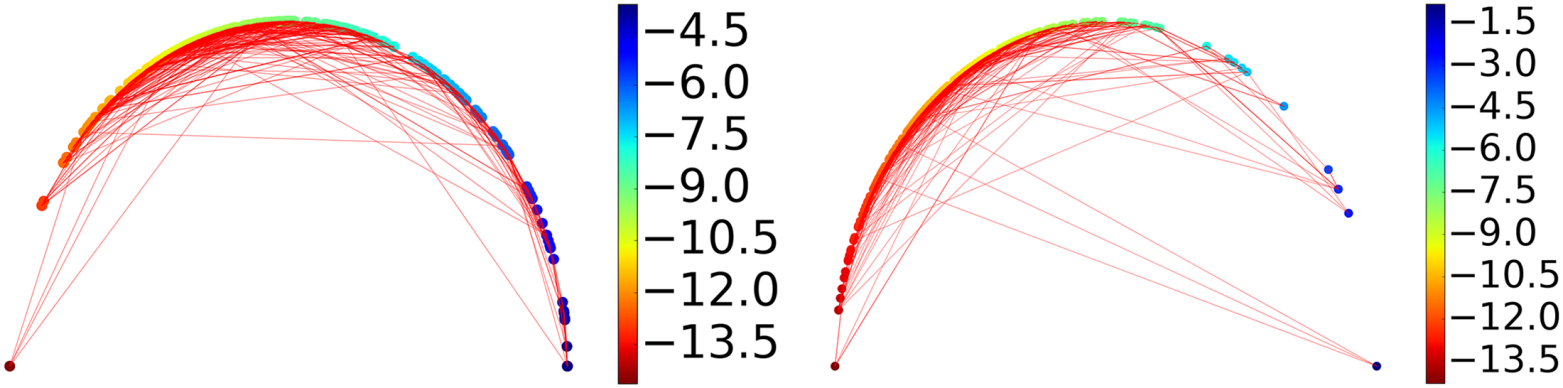
An illustration of similarities between ligands measured by their barcode space Wasserstein distances. Ligands are ordered according to their binding affinities and are represented as dots on the semicircle. Specifically, a sample of binding free energy *x* is plotted at the angle *θ* = *π*(*E*_*max*_ − *x*)/(*E*_*max*_ − *E*_*min*_) where *E*_*min*_ and *E*_*max*_ are the lowest and the highest energy in the dataset. Each dot is connected with two nearest neighbors based on their barcode space Wasserstein distances. An optimal prediction would be achieved if lines stay close to the semicircle. The majority of the connections stay near the boundary to the upper half sphere demonstrating that barcode space metric based Wasserstein distance measurement reflects the similarity in function, i.e., the binding affinity in this case. The protein clusters with the best and the worst performance are shown. Left: Protein cluster 2. Right: Protein cluster 3.

The barcodes generated using Rips complex with distance matrices **M** and ${\tilde{M}}^{1}$ are collected and the distance between each pair of barcodes are measured using the Wasserstein metric *d*^2^. Leave-one-out prediction for every sample is performed with k-nearest neighbor regression with *k* = 3 within each protein cluster based on the Wasserstein metric. The results are shown in S1 Text Table C. The performance of the best performing and the worst performing protein clusters is shown in Fig 3. The better the performance, the closer the lines are to the semicircle.

The experiments done for this section are summarized in Table 7.

**Table 7:** Experiments for ligand-based protein-ligand binding affinity prediction of 7 protein clusters and 1322 protein-ligand complexes.

Experiment	Description
A-B012-E-C-GBT	The barcodes are generated using alpha complex on different sets of atoms based on different element combinations. The features are constructed using the 0th, 1st, and 2nd dimensional barcodes following the *counts in bins* method with bins equally dividing the interval [0, 5]. Here 32 different element combinations are considered, including {C, N, O, S, CN, CO, CS, NO, NS, OS, CNO, CNS, COS, NOS, CNOS, CNOSPFClBrI, H, CH, NH, OH, SH, CNH, COH, CSH, NOH, NSH, OSH, CNOH, CNSH, COSH, NOSH, CNOSH, CNOSPFClBrIH}. Gradient boosting trees (GBT) with the structured feature matrix are used for this computation.
A-B012-E-S-GBT	The barcodes same as those used in A-B012-E-C-GBT are used. Instead of *counts in bins*, the *Barcode statistics* method is used to generate features.
A-B012-E-SS-GBT	The barcodes same as those used in A-B012-E-C-GBT are used. The *persistence diagram slice and statistics* method is used to generate features. A uniform set of bins by dividing the interval [0, 5] into 10 equal length bins is used to slice birth, death, and persistence values.
R-B012-E-S-GBT	Barcodes are generated using Rips complex with Euclidean distances. The features are generated following the *barcode statistics* method. Here 36 element combinations are considered, i.e., {C, N, O, S, CN, CO, CS, NO, NS, OS, CNO, CNS, COS, NOS, CNOS, CNOSPFClBrI, H, CH, NH, OH, SH, CNH, COH, CSH, NOH, NSH, OSH, CNOH, CNSH, COSH, NOSH, CNOSH, CNOSPFClBrIH, CCl, CClH, CBr, CBrH}.
R-B012-M1-S-GBT	The result is obtained with the same setup as R-B012-E-S-GBT except that the first level enrichment distance matrix ${\tilde{M}}^{1}$ is used instead of Euclidean distance.
R-B*n*-E-KNN	The *n*th dimensional barcodes from Rips complex computation with Euclidean distance are used. K-nearest neighbor (KNN) regression is performed with Wasserstein metric *d*^2^. The leave-one-out validation is performed individually with each element combination and the average prediction of these element combinations is taken as the output result. The element combinations considered are {CNOS, CNOSPFClBrI, NOH, CNO, CNOSPFClBrIH}. These combinations are selected based on their performance in the gradient boosting trees experiments.
R-B*n*-M1-KNN	The result is obtained with the same setup as R-B*n*-E-KNN except that the distance matrix ${\tilde{M}}^{n}$ is used instead of Euclidean distance.

#### Performance of multi-component persistent homology

It can be noticed from Table 8 that topological features generated from barcode statistics typically outperform those created from counts in bins. R-B012-E-S-GBT and R-B012-M1-S-GBT perform similarly in the majority of the protein clusters whilst R-B012-M1-S-GBT which is based on ${\tilde{M}}^{1}$ significantly outperforms R-B012-E-S-GBT which is based on Euclidean distance in protein cluster 3 and 6. To assess in what circumstances does the multi-level persistent homology improve the original persistent homology characterization of small molecules, we analyze the statistics of the size of ligands in Fig 2. It turns out that protein cluster 3 has the smallest average number of heavy atoms and protein cluster 6 has the smallest standard deviation of the number of heavy atoms. This observation partially answers the question that in the cases where the small molecules are relatively simple and are relatively of similar size, multi-level persistent homology is able to enrich the characterization of the small molecules which further improves the robustness of the model. Such enrichment or improvement over the original persistent homology approach is mainly realized in higher dimensional barcodes, i.e. the 1st and 2nd dimensions. In Table 8, the results with ID through 7 to 12 confirm that the 0th dimensional features from computation with ${\tilde{M}}^{1}$ are inferior to the results with Euclidean distance whilst the 1st and 2nd dimensional features based on ${\tilde{M}}^{1}$ outperforms the best result with Euclidean distance in most cases.

**Table 8:** Performance of different approaches on the S1322 dataset.

ID	Experiments	CL 1 (333)	CL 2 (264)	CL 3 (219)	CL 4 (156)	CL 5 (134)	CL 6 (122)	CL 7 (94)	Average
1	A-B012-E-C-GBT	0.695(1.63)	0.836(1.18)	0.690(1.52)	0.642(1.38)	**0.840(1.30)**	0.647(1.65)	0.730(1.27)	0.726(1.42)
2	A-B012-E-S-GBT	0.695(1.63)	0.845(1.14)	0.678(1.54)	**0.692(1.31)**	0.828(1.35)	0.702(1.54)	0.739(1.25)	0.740(1.39)
3	A-B012-E-SS-GBT	0.704(1.62)	0.846(1.15)	0.681(1.53)	0.668(1.35)	0.834(1.34)	0.715(1.53)	0.741(1.25)	0.741(1.40)
4	R-B012-E-S-GBT	0.712(1.60)	0.837(1.17)	0.659(1.57)	0.683(1.32)	0.808(1.41)	0.635(1.67)	**0.757(1.22)**	0.727(1.42)
5	R-B012-M1-S-GBT	**0.716(1.59)**	0.836(1.17)	**0.706(1.48)**	0.672(1.34)	0.822(1.37)	**0.708(1.53)**	0.746(1.24)	0.744(1.39)
6	2+5	0.714(1.59)	**0.848(1.13)**	0.699(1.50)	**0.692(1.31)**	0.831(1.34)	**0.717(1.52)**	0.747(1.24)	**0.750(1.38)**
7	R-B0-E-KNN	0.648(1.73)	0.761(1.39)	0.544(1.76)	0.616(1.42)	0.700(1.70)	0.487(1.89)	0.641(1.43)	0.628(1.62)
8	R-B1-E-KNN	0.547(1.91)	0.684(1.55)	0.444(1.88)	0.536(1.52)	0.535(2.01)	0.634(1.67)	0.649(1.42)	0.576(1.71)
9	R-B2-E-KNN	0.474(2.01)	0.494(1.87)	0.202(2.14)	0.298(1.79)	0.126(2.49)	0.331(2.09)	0.609(1.47)	0.362(1.98)
10	R-B0-M1-KNN	0.581(1.85)	0.771(1.35)	0.516(1.80)	0.601(1.44)	0.672(1.76)	0.485(1.90)	0.644(1.43)	0.610(1.65)
11	R-B1-M1-KNN	0.663(1.70)	0.784(1.33)	0.652(1.59)	0.555(1.50)	0.786(1.49)	0.610(1.71)	0.731(1.30)	0.683(1.52)
12	R-B2-M1-KNN	0.675(1.67)	0.803(1.28)	0.577(1.72)	0.531(1.52)	0.655(1.81)	0.617(1.72)	0.648(1.42)	0.644(1.59)
13	Cons(7+8+9+10+11+12)	0.698(1.66)	0.817(1.28)	0.620(1.68)	0.645(1.41)	0.756(1.68)	0.658(1.68)	0.739(1.31)	0.705(1.49)
14	2+5 (5-fold)	0.713(1.60)	0.843(1.15)	0.693(1.51)	0.670(1.35)	0.831(1.34)	0.698(1.56)	0.737(1.26)	0.741(1.40)

Pearson correlation coefficients with RMSE (kcal/mol) in parentheses for binding affinity predictions on 7 protein clusters (CL) in S1322. On the title row, the numbers in parentheses denote the numbers of ligands in the cluster. The median results of 20 repeated runs are reported for the ensemble of trees based methods to account for randomness in the algorithm. For experimental labels, the first letter indicates the complex definition used, ‘A’ for alpha complex and ‘R’ for Rips complex. The second part starting with ‘B’ followed by the integers indicates the dimension of barcode used. The third part indicates the distance function used, ‘E’ for Euclidean and ‘M1’ for ${\tilde{M}}^{1}$. For row 1 through 5, the forth part shows the way of feature construction, ‘C’ for counts in bins and ‘S’ for barcode statistics. The last part indicates the regression technique used, ‘GBT’ for gradient boosting trees and ‘KNN’ for k-nearest neighbors. The detailed descriptions of the experiments are given in Table 7. Row 6 is the results using features of both row 2 and row 5. Row 13 is the consensus results by taking the average of the predictions by row 7 through row 12. Except for specified, all results are obtained from 10-fold cross validations.

It is interesting to note that although Wasserstein metric based KNN methods are not as accurate as GBT approaches, the consensus result obtained by averaging over various predictions with Wasserstein metric on different sets of barcodes is quite accurate.

#### Robustness of topological learning models

**Fig 4:**
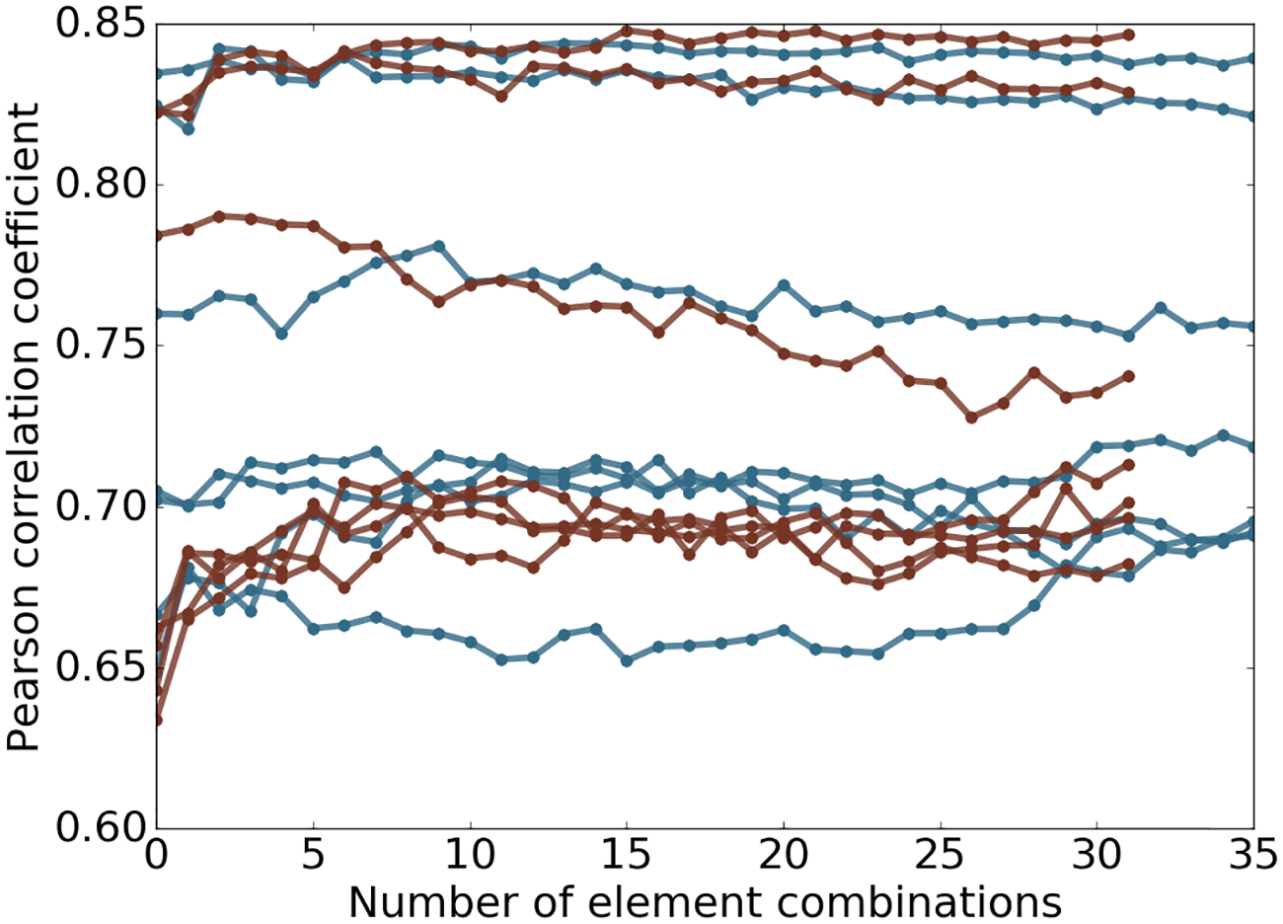
Plot of performance against number of element combinations used. The topological learning model performance against the number of element combinations involved in feature construction for 7 protein clusters in S1322. The horizontal axis corresponds to the number of element combinations used for the features. From left to right, one extra element combination is added at a step. The features are then used in gradient boosting trees method to test if the model is robust against redundant information. The results related to alpha complex are marked in red and Rips complex in blue. The median Pearson correlation coefficient between predicted and experimental results is reported of 10-fold cross-validation within each protein cluster repeated 20 times are reported.

Certain elements such as Br are very rare in the data sets studied in this work. Considering only the elements of high occurrence will not hurt the performance on the validations performed. However, omitting the low occurrence elements will sacrifice the capability of the model to handle new data in which such elements play an important role. Therefore, we decide to keep the rare elements that result in a large number of features and redundancy in features. For example, the element combinations CBrH and CH will probably deliver the same performance for most of the samples in the data sets studied in this work. To test whether this redundancy causes degenerated results of the model, the features of one element combination is added to the model at a step and the model is validated with an accumulation of the added features at each step. The performance of the model is measured with Pearson correlation coefficient and is plotted against number of element combinations involved in Fig 4. For most cases in Fig 4, the model is robust against the inclusion of more element combinations.

### Complex based protein-ligand binding affinity prediction

Having demonstrated the representational power of the present topological learning method for characterizing small molecules, we further examine the method on the task of characterizing protein-ligand complex. Biologically, we consider the same task, i.e., the prediction of protein-ligand binding affinity, with a different approach that is based on the structural information of the protein-ligand complexes. Only gradient boosting trees and deep convolutional neural network algorithms are used in this section. All the gradient boosting trees models take the setup described in *Section Methods/Machine learning algorithms/Gradient boosting trees*.

In the present topological learning study, we use four versions of PDBBind core sets as our test sets. For each test set, the corresponding refined set, excluding the core set, is used as the training set.

#### Groups of topological features and their performance in association with GBT

The experiments of protein-ligand-complex-based protein-ligand binding affinity prediction for the PDBBind datasets are summarized in Table 9.

**Table 9:** Experiments for protein-ligand-complex-based protein-ligand binding affinity prediction for the PDBBind datasets.

Experiment	Description
R-B0-I-C	0th dimensional barcodes from Rips complex computation with interactive distance matrix based on Euclidean distance are used. Features are generated following *counts in bins* method with bins {[0, 2.5), [2.5, 3), [3, 3.5), [3.5, 4.5), [4.5, 6), [6, 12]}. Element combinations used are all possible paired choices of one item from {C, N, O, S, CN, CO, NO, CNO} in protein and another item from {C, N, O, S, P, F, Cl, Br, I, CN, CO, CS, NO, NS, OS, CNO, CNS, COS, NOS, CNOS} in ligand, which result in a total of 160 combinations.
R-B0-I-BP	The persistent homology computation and feature generation is the same as R-B0-I-C. However, the element combinations used are all possible paired choices of one item from {C, N, O, S} in protein and another item from {C, N, O, S, P, F, Cl, Br, I} in ligand, which result in a total of 36 element combinations.
R-B0-CI-C	0th dimensional barcodes from Rips complex computation with interactive distance matrix based on the electrostatics correlation function defined in Eq (10) with the parameter *c* = 100. The features are generated following *counts in bins* method with bins {(0, 0.1], (0.1, 0.2], (0.2, 0.3], (0.3, 0.4], (0.4, 0.5], (0.5, 0.6], (0.6, 0.7], (0.7, 0.8], (0.8, 0.9], (0.9, 1.0)}. The element combinations used are all possible paired choices of one item from {C, N, O, S, H} in protein and another item from {C, N, O, S, P, F, Cl, Br, I, H} in ligand, which result in a total of 50 element combinations.
R-B0-CI-B-S	The barcodes and element combinations are the same as those of R-B0-CI-B-C. The features are generated following the *barcode statistics* method.
A-B12-E-S	1st and 2nd dimensional barcodes from alpha complex computation with Euclidean distance are used. The element combinations considered are all heavy atoms and all carbon atoms. Features are generated following the *barcode statistics* method.

#### Robustness of GBT algorithm against redundant element combination features and potential overfitting

It is intuitive that combinations of more than 2 element types are able to enrich the representation especially in the case of higher dimensional barcodes. However, the consideration of combination of more element types rapidly increases the dimension of feature space. In the high dimensional feature space, it is almost inevitable that there exists nonessential and redundant features. Additionally, the importance of a feature varies across different problems and data sets. Therefore, it is preferable to keep all the potentially important features in a general model which is expected to cover a wide range of situations. To test the robustness of the model against unimportant features, we select a total of 128 element combinations (i.e., all possible paired choices of one item from {C, N, O, CN, CO, NO, CNO, CNOS} in protein and another item from {C, N, O, S, CN, CO, CS, NO, NS, OS, CNO, CNS, COS, NOS, CNOS, CNOSPFClBrI} in ligand). The 0th, 1st, and 2nd dimensional barcodes are computed for all combinations using alpha complex with Euclidean distance. Features are generated following the barcode statistics method.

A general model with all the features is generated in the first place. The element combinations are then sorted according to their importance scores in the general model. Starting from the most important element combination, one element combination is added to the feature vector each time and then the resulting feature vector is passed to the machine learning training and testing procedure. The order of adding element combinations is based on their importance scores and thus that a less important feature is added each step.

**Fig 5:**
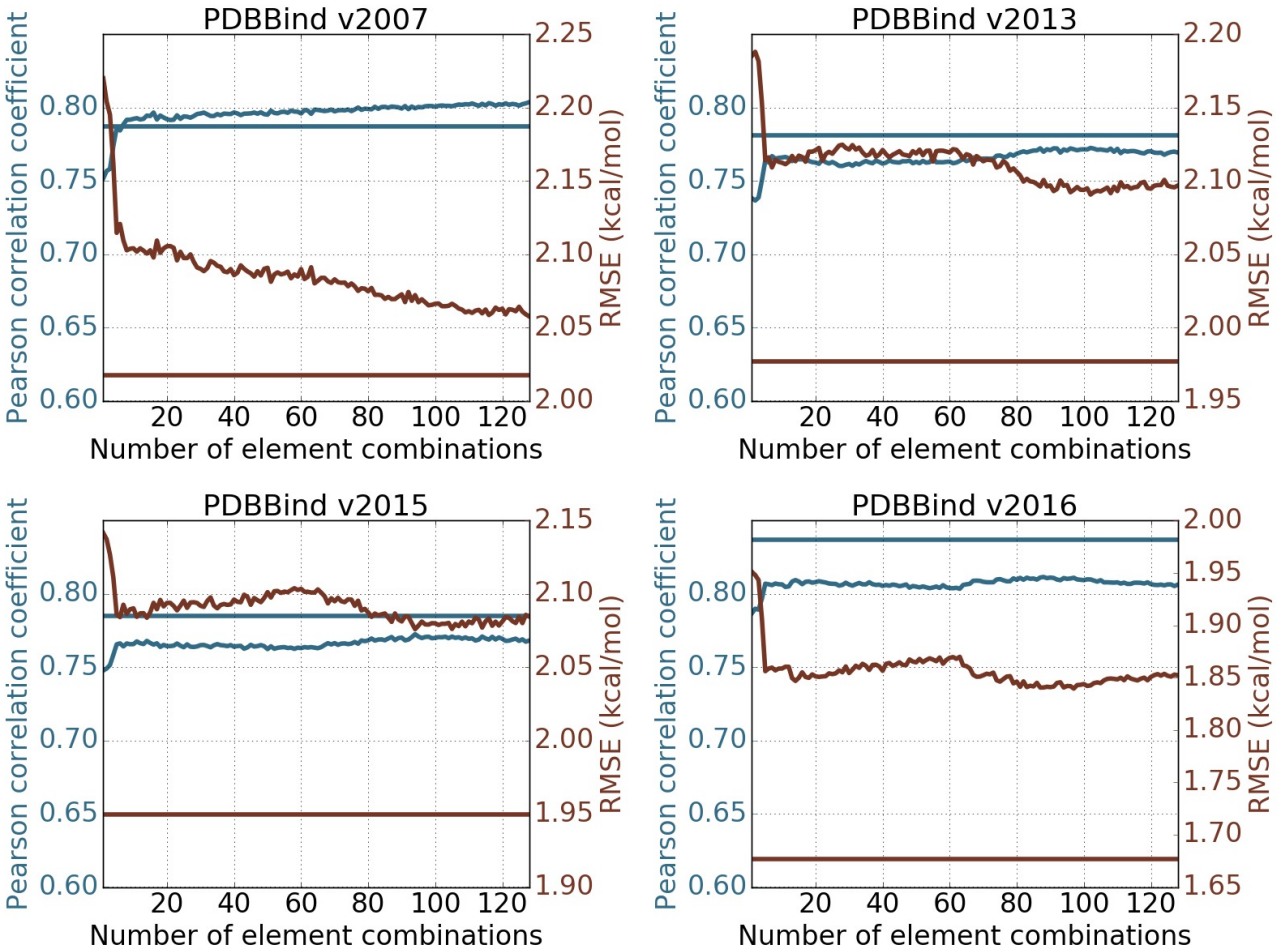
Feature robustness tests on PDBBind datasets. The performance of the topological learning model against the number of included element combinations for predicting on PDBBind core sets and training on PDBBind refined sets minus the core sets. The 1st and 2nd dimensional barcodes computed with alpha complex is used. Features are generated following *barcode statistics* method. Element combinations are all possible paired choices of one item from {C, N, O, CN, CO, NO, CNO, CNOS} in protein and another item from {C, N, O, S, CN, CO, CS, NO, NS, OS, CNO, CNS, COS, NOS, CNOS, CNOSPFClBrI} in ligand, which result in 128 element combinations. The horizontal straight lines represents the performance of the 2D representation with deep convolutional neural network (row 10 in Table 10). The blue and red colors correspond to Pearson correlation coefficient and RMSE (kcal/mol) respectively. Each experiment is done by training on refined set minus the core set with the median result of 20 repeated runs reported.

Fig 5 depicts the changes of Pearson correlation coefficient and RMSE (kcal/mol) with respect to the increase of element combinations in predicting four PDBBind core sets. In all cases, the inclusion of top combinations can readily deliver very good models. The behavior of the present method in PDBBind v2007 is quite different from that in other data sets. The performance of the present method improves almost monotonically as the element combination increases. However, in other three cases, the improvement is unsteady. Nevertheless, the performance fluctuates within a small range, which indicates that the present method is reasonably stable against the increase in element combinations. From a different perspective, the increase in element combinations might lead to overfitting in machine learning. Since the model parameters are fixed before the experiments, it shows that GBT algorithms are not very sensitive to redundant features and are robust against overfitting.

#### Usefulness of more than 2 element types for interactive 0th dimensional barcodes

While using element combinations with more than 2 element types with higher dimensional barcodes enriches characterization of geometry, it remains to assess whether interactive 0th dimensional characterization will benefit from element combinations with more element types. As an example, we denote interactive 0th dimensional barcodes for carbon and nitrogen atoms from protein and oxygen atoms from ligand by **B**_CN−O_, barcodes for carbon atoms from protein and oxygen atoms from ligand by **B**_C−O_, and barcodes for nitrogen atoms from protein and oxygen atoms from ligand by **B**_N−O_. In the case of persistent homology barcode representation, **B**_CN−O_ is not strictly the union of **B**_C−O_ and **B**_N−O_. However **B**_CN−O_ might be redundant to **B**_C−O_ and **B**_N−O_. To address this concern, we test features from interactive 0th dimensional barcodes with the 36 element combinations (i.e., {C, N, O, S} for protein and {C, N, O, S, P, F, Cl, Br, I} for ligand) and features for the 160 selected element combinations (i.e., {C, N, O, S, CN, CO, NO, CNO} for protein and {C, N, O, S, P, F, Cl, Br, I, CN, CO, CS, NO, NS, OS, CNO, CNS, COS, NOS, CNOS} for ligand), which are listed as feature group 2 and feature group 1 in Table 10. In all the four cases, the features of the 36 combinations (feature group 2) slightly outperforms or performs as well as the features of the 160 combinations (feature group 1) suggesting that element combinations with more than 2 element types are redundant to all the combinations with 2 element types in the case of interactive 0th dimensional characterization.

**Table 10:** Performance of different protein-ligand complex based approaches on the PDBBind datasets.

ID	Experiments	v2007	v2013	v2015	v2016	Average
1	R-B0-I-C	0.799 (2.01)	0.741 (2.14)	0.750 (2.11)	0.813 (1.82)	0.776 (2.02)
2	R-B0-I-BP	**0.816 (1.94)**	0.741 (2.13)	0.750 (2.10)	0.825 (1.78)	0.783 (1.99)
3	R-B0-CI-C	0.791 (2.05)	0.759 (2.10)	0.738 (2.13)	0.801 (1.87)	0.772 (2.04)
4	R-B0-CI-S	0.773 (2.10)	0.762 (2.12)	0.749 (2.13)	0.810 (1.86)	0.774 (2.05)
5	A-B12-E-S	0.736 (2.25)	0.709 (2.26)	0.695 (2.27)	0.752 (2.02)	0.723 (2.20)
6	1+4	0.815 (1.95)	0.780 (2.04)	0.774 (2.04)	0.833 (1.76)	0.801 (1.95)
7	2+4	0.806 (1.99)	0.787 (2.04)	0.770 (2.06)	0.834 (1.77)	0.799 (1.97)
8	1+4+5	0.810 (1.98)	0.792 (2.02)	0.786 (2.02)	0.831 (1.76)	0.805 (1.95)
9	2+4+5	0.802 (2.01)	**0.796** (2.02)	0.782 (2.04)	0.822 (1.79)	0.801 (1.97)
10	2D-CNN-Alpha	0.787 (2.02)	0.781 (**1.98**)	0.785 (1.95)	0.837 (1.68)	0.798 (1.91)
11	1D2D-CNN	0.806 (1.95)	0.781 (**1.98**)	**0.799 (1.91)**	**0.848 (1.64)**	**0.809 (1.87)**

Pearson correlation coefficients with RMSE (kcal/mol) in parentheses for predictions by various groups of features on the four PDBBind core sets. The training sets are the PDBBind refined sets minus the core sets of the same version year. Results of ensemble of trees based methods (rows 1 through 9) are the *median values* of 50 repeated runs to account for randomness in the algorithm. For the deep learning based methods (row 10 and 11), 100 independent models are generated in the first place. A consensus model is built by randomly choosing 50 models out of the 100, and the this process is repeated 1000 times with the median reported. The first letter indicates the definition of complex, ‘A’ for alpha complex and ‘R’ for Rips complex. The second part indicates the dimension of barcodes used. The third part indicates the distance function used, ‘I’ for ${\hat{M}}_{ij}$ defined in Eq (5), ‘CI’ for the one defined in Eq (10), and ‘E’ for Euclidean. The last part shows the way of feature construction, ‘C’ for counts in bins, ‘S’ for barcode statistics, and ‘BP’ for only pair of two single elements. The results reported in row 6 through 9 are obtained by combining the features of the rows with the corresponding numbers.

#### Importance of atomic charge in electrostatic persistence

In element specific persistent homology, atoms of different element types are characterized separately, which offers a rough and implicit description of the electrostatics of the system. However, such implicit treatment of electrostatics may lose important information because atoms behave differently at different oxidation states. Therefore, we explicitly embed atomic charges in interactive 0th dimensional barcodes as described in Eq (10). The resulting topological features are given in feature group 4 in Table 10. It can be seen from Table 10 that the combination of feature group 4 and the Euclidean distance based interactive 0th dimensional barcodes (listed as feature group 6 and 7) generally outperforms the results obtained with only Euclidean distance based features. This observation suggests that electrostatics play an important role and should be taken care of explicitly for the protein-ligand binding problem. Additionally, the inclusion of physical interactions in topological invariants opens a promising new direction in topological analysis.

#### Relevance of elements that are rare with respect to the data sets

**Fig 6:**
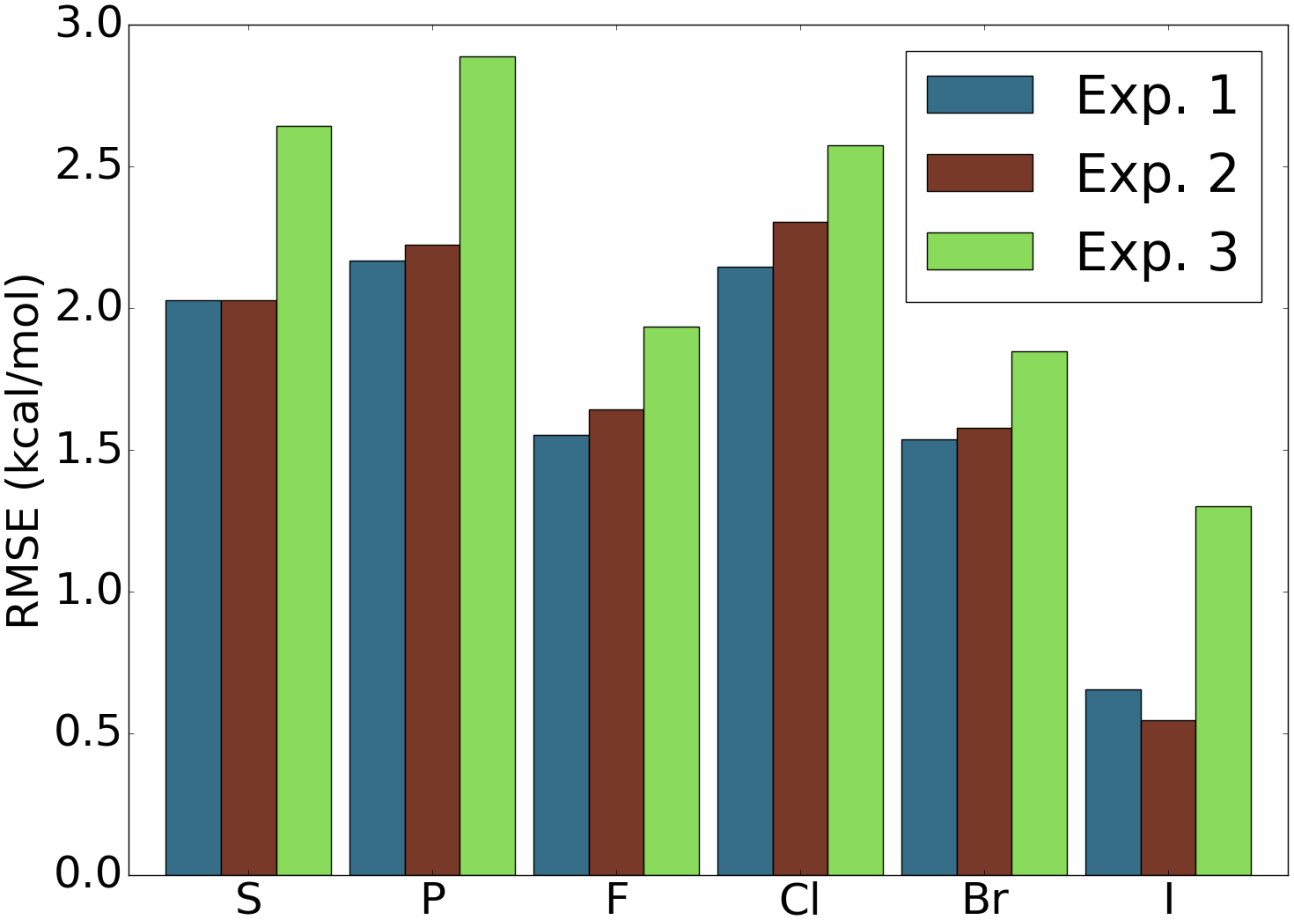
Assessment of performance of the model on samples with elements that are rare in the data sets. For the four data sets PDBBind v2007, v2013, v2015, and v2016 [99], and for each element, the testing set is the subset of the original core sets with only ligands that contain atoms of the particular element type. The features used are features with ID = 7 in Table 10. The reported RMSE is the average taken over the four data sets. Experiment 1: Training set is the original training set and all the features are used. Experiment 2: Training set is the original training set and only features that do not involve the particular element are used. Experiment 3: Training set is the original training set excluding the samples that contain atoms of the particular element type and all features are used. For most of the elements, experiment 1 achieves the best result and experiment 3 yields the worst performance.

Since the majority of the samples in both training and testing sets only contain atoms of element types, C, N, O, and H, the performance of the model on the samples with rare occurring elements with respect to data sets is hardly reflected by the overall performance statistics. For simplicity, we refer to such rarely occurring elements with respect to data sets simply by rarely occurring elements in the discussion follows. To assess the aspects of the model that potentially affect the performance on the samples containing rarely occurring elements, we picked the samples containing each rarely occurring element from the original testing set as a new testing set. Three experiments are carried out to address two questions: “Are the training samples containing the same rarely occurring element crucial?” and “Are features addressing the rarely occurring element important?”. A short answer is yes to both according to the results shown in Fig 6. Specifically, for each rarely occurring element, the exclusion of samples containing this element in training set and the exclusion of features addressing this element will both cause degenerated results. It is also shown that the exclusion of samples of the rarely occurring element leads to much worse results. Since both modifications of the model deliver worse results, we conclude that including the samples in the training set with similar compositions to the test sample is crucial to the success of the model on this specific test sample. Even the inclusion of features of more element types or element combinations does not deliver better results in the general testing sets, such features should still be kept in the model in case that a sample with a similar element composition comes in as a test sample.

#### 2D persistence for topological deep convolutional neural networks

**Fig 7:**
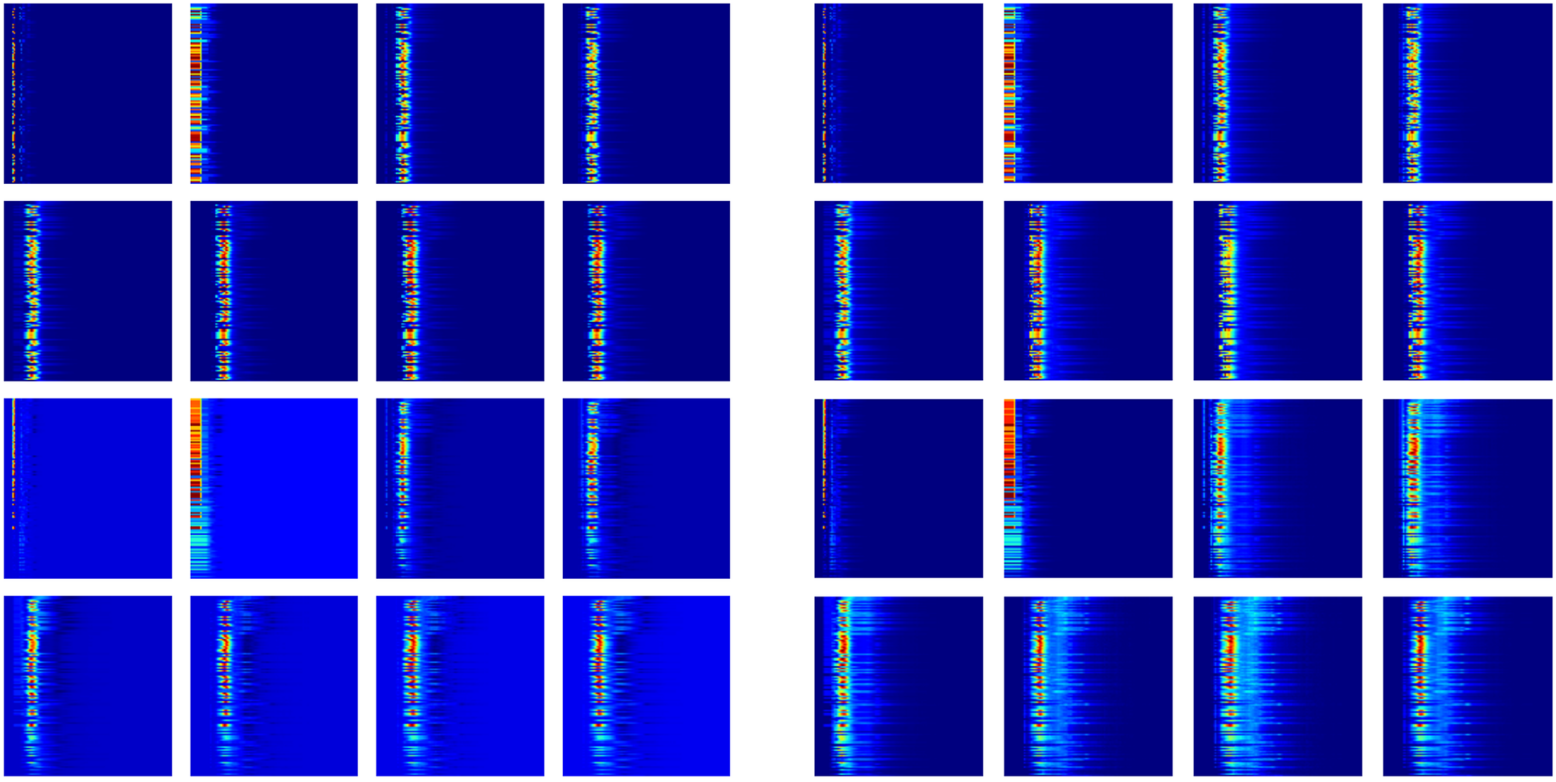
Heat map plot of the 16 channels. The mean value (left image) and the standard deviation (right image) of each digit over the PDBBind v2016 refined set are shown. The top 8 maps are for protein-ligand complex and the other 8 maps are for the difference between protein-ligand complex and protein only. For each map, the vertical axis is the element combinations ordered according to their importance and the horizontal axis is the dimension of spatial scales.

Deep learning is potentially more powerful than many other machine learning algorithms when the data size is sufficiently large. In the present work, it is natural to construct a 2D topological representation by incorporating the element combination as an additional dimension, resulting in 16 channels as defined in *Section Feature generation from topological invariants*. Here 128 element combinations (i.e., all possible paired choices of one item from {C, N, O, CN, CO, NO, CNO, CNOS} in protein and another item from {C, N, O, S, CN, CO, CS, NO, NS, OS, CNO, CNS, COS, NOS, CNOS, CNOSPFClBrI} in ligand) are used for 2D analysis. The advantage of introducing this extra dimension with convolutional neural networks is to prevent unimportant features from interacting with important ones at the lower levels of the model whilst generally unimportant features are still kept in the model in case that they are essential to specific problems or a certain portion of the data set. Fig 7 illustrates the mean value and the standard deviation of the PDBBind v2016 refined set. The existence of significant standard deviations for relatively unimportant element combinations indicates that these features might still contribute to the overall prediction.

As shown in Fig 5, for all the data sets except the PDBBind v2007 set, the 2D topological deep learning with convolutional neural networks performs significantly better. The inferior performance of convolutional neural networks in v2007 might be a result of the small data size. Note that v2007 training set has 1105 protein-ligand complexes, whereas other training sets have more than 2700 complexes. Consequently, topological deep convolutional neural networks are able to outperform the topological GBT algorithm in predicting v2013, v2015 and v2016 core sets. Indeed, topological deep convolutional neural networks have advantages in dealing with large data sets.

### Structure-based virtual screening

In our final model TopVS reported in Table 6, we use topological descriptors of both protein-compound interactions and only the compounds (i.e., ligands and decoys) and take a consensus model on top of several ensemble of trees models and a deep learning model. We have also tested the behavior of our topological learning model TopVS-ML using either one of the aforementioned descriptions. The tests are done with TopVS-ML because that TopVS-DL is much more time consuming. When only topological descriptor of small molecules are used, which falls into the category of ligand-based virtual screening, an AUC of 0.81 is achieved. For the topological learning model using only the descriptions of protein-ligand interactions, an AUC of 0.77 is achieved. An AUC of 0.83 is obtained with a model combining both sets of descriptors which is better than each individual performance, suggesting that the two groups of descriptors are complementary to each other and are both important for achieving satisfactory results. The marginal improvement made by protein-compound complexes maybe due to the various docking quality. Similar situation was encountered by a deep learning method [98]. For the targets with high quality results by Autodock Vina (AUC of ADV > 0.8), the ligand-based features achieve an AUC of 0.81 and the complex-based features achieve an AUC of 0.86. On the other hand, for the targets with low quality results by Autodock Vina (AUC of ADV < 0.5), the ligand-based features achieve an AUC of 0.82 and the complex-based features achieve an AUC of 0.74. The results of these cases are listed in S1 Text, Tables H and I. This observation suggests that the performance of features describing the interactions and the geometry of protein-compounds complexes highly depends on the quality of docking results.

Our model with small molecular descriptors delivers an AUC of 0.81, which is comparably well to the other top performing methods. The performance of this model is also competitive in the regime of protein-ligand binding affinity prediction based on experimentally solved complex structures as is shown in *Section Discussion/Ligand based protein-ligand binding affinity prediction*. These results suggest that topology based small molecule characterization proposed in this work is potentially useful in other applications involving small molecules, such as predictions of toxicity, solubility and partition coefficient of small molecules.

### Conclusion

Persistent homology is a relatively new branch of algebraic topology and is one of the main tools in topological data analysis. The topological simplification of biomolecular systems was a major motivation of the earlier persistent homology development [29, 36]. Persistent homology has been applied to computational biology [76, 77, 77–79], including our efforts [26, 87–91, 93]. However, the predictive power of primitive persistent homology was limited in early topological learning applications [92]. To address this challenge, we have recently introduced element specific persistent homology to retain chemical and biological information during the topological abstraction of biomolecules [14, 27, 94]. The resulting topological learning approach offers competitive predictions of protein-ligand binding affinity and mutation induced protein stability changes. However, persistent homology based approaches for small molecules have not been developed and its representability and predictive powers for the interaction of small molecules with macromolecules have not been extensively studied.

The present work further introduces multi-component persistent homology, multi-level persistent homology and electrostatic persistence for chemical and biological characterization, analysis and modeling. Multi-component persistent homology takes a combinatorial approach to create possible element specific topological representations. Multi-level persistent homology allows tailored topological descriptions of any desirable interaction in biomolecules which is especially useful for small molecules. Electrostatic persistence incorporates partial charges that are essential to biomolecules into topological invariants. These approaches are implemented via the appropriate construction of the distance matrix for filtration. The representation power and reduction power of multi-component persistent homology, multi-level persistent homology and electrostatic persistence are validated by two databases, namely PDBBind [99] and DUD [107, 108]. PDBBind involves more than 4,000 high quality protein-ligand complexes and DUD contains 128,374 compound-target pairs. Two classes of problems are used to test the proposed topological methods, including the prediction of protein-ligand binding affinities and the discrimination of active ligands from decoys (virtual screening). In both problems, we examine the representability of proposed topological learning methods on small molecules, which are somewhat more difficult to describe by persistent homology due to their chemical diversity, variability and sensitivity. Additionally, these methods are tested on their ability to handle the full protein-ligand complexes. Advanced machine learning methods, including Wasserstein metric based k-nearest neighbors (KNNs), gradient boosting trees (GBT), random forest (RF), extra trees (ET) and deep convolutional neural networks (CNN) are utilized in the present work to facilitate the proposed topological methods, rendering advanced topological learning algorithms for quantitative and qualitative biomolecular predictions. The thorough examination of the method on the prediction of binding affinity for experimentally solved protein-ligand complexes leads to a structure-based virtual screening method, TopVS, which outperforms other methods. The feature sets introduced in this work for small molecules and protein-ligand complexes can be extended to other applications such as 3D-structure based prediction of toxicity, solubility, and partition coefficient for small molecules and complex structure based prediction of protein-nucleic acid binding and protein-protein binding affinities.

## Methods

### Persistent homology

The concept of persistent homology is built on the mathematical concept of homology, which associates a sequence of algebraic objects, such as abelian groups, to topological spaces. For discrete data such as atomic coordinates in biomolecules, algebraic groups can be defined via simplicial complexes, which are constructed from simplices, generalizations of the geometric notion of nodes, edges, triangles and tetrahedrons to arbitrarily high dimensions. Homology characterizes the topological connectivity of geometric objects in terms of topological invariants, i.e., Betti numbers, which are used to distinguish topological spaces by counting *k*-dimensional holes. Betti-0, Betti-1 and Betti-2, respectively, represent independent components, rings and cavities in a physical sense. In persistent homology, the generators in the homology groups are tracked along with a filtration parameter, such as the radius of a ball or the level set of a hypersurface function, that continuously varies over a range of values. Therefore, persistent homology is induced by the filtration. For a given biomolecule, the change and the persistence of topological invariants over the filtration offer a unique characterization. These concepts are very briefly discussed below. For more detailed theory and algorithms, the interested readers are referred to a book on computational topology [117].

#### Simplicial complex

A (geometric) *k-simplex* denoted *σ*^*k*^ is the convex hull of *k* + 1 affinely independent points in $R^{k}$. The convex hull of each nonempty subset of the *k* + 1 points forms a subsimplex and is regarded as a *face* of *σ*^*k*^. The points are also called *vertices* of *σ*^*k*^.

A set of simplices *K* is a *simplicial complex* if all faces of any simplex in *K* are also in *K* and the intersection of any pair of simplices in *K* is either empty or a common face of the two simplices.

#### Homology

A *k-chain* of a simplicial complex *K* denoted by *c*_*k*_ is a formal sum of *k-simplices* in *K*. Here, we take the $Z_{2}$ field for the coefficients of the formal sum. Under the addition operation of $Z_{2}$, a group of *k-chains* is called a *chain group* and denoted *C*_*k*_(*K*) which has the basis as the set of *k-simplices* in *K*.

A *boundary operator* denoted by ∂_*k*_: *C*_*k*_(*K*) → *C*_*k*−1_(*K*) maps a *k-chain* which is a linear combination of *k-simplices* to the same linear combination of the boundaries of the *k-simplices*. With a *k-simplex* *σ*^*k*^ = [*v*_0_, …, *v*_*k*_] where *v*_*i*_ are the vertices of *σ*^*k*^, the *boundary operator* is defined as $\partial_{k}\sigma^{k}=\sum_{i=0}^{k}\sigma_{i}^{k-1}$, where $\sigma_{i}^{k-1}$ is a *(k-1)-simplex* which is a face of *σ*^*k*^ with the *i*th vertex being absent. Since we are working with the $Z_{2}$ coefficients, we omit the orientations of the simplices.

A *k-cycle* is a *k-chain* whose image under the *boundary operator* ∂_*k*_ is the empty set. The collection of all the *k-cycles* forms a group denoted *Z*_*k*_(*K*) which is the kernel of ∂_*k*_: *C*_*k*_(*K*) → *C*_*k*−1_(*K*).

The image of ∂_*k*+1_: *C*_*k*+1_(*K*) → *C*_*k*_(*K*) is called the boundary group and is denoted by *B*_*k*_(*K*). *B*_*k*_(*K*) is a subgroup of *Z*_*k*_(*K*) following the property of the *boundary operator* that ∂_*k*_ ∘ ∂_*k*+1_ = 0.

The *k*th *homology group* is the quotient group defined as *H*_*k*_(*K*) = *Z*_*k*_(*K*)/*B*_*k*_(*K*). Its ranks are the Betti numbers of *K* and its generators (equivalence classes) are also of interest.

The *k*th *Betti number* *β*_*k*_ is defined and often computed as rank*H*_*k*_(*K*) = rank*Z*_*k*_(*K*) − rank*B*_*k*_(*K*). Intuitively, Betti numbers count the number of *k*-dimensional holes that can not be continuously deformed to each other. Analogous to the continuous case, in simplicial topology, two cycles (elements of *Z*_*k*_(*K*)) that defer by the boundary of a chain (an element of *B*_*k*_(*K*)) are considered to be able to deform continuously to each other and are thus representing the same element in *H*_*k*_(*K*).

#### Persistent homology

A *filtration* of a *simplicial complex* *K* is a nested sequence of subcomplexes of *K* such that ⌀ = *K*^0^ ⊂ *K*^1^ ⊂ … ⊂ *K*^m^ = *K*. Each *K*^*i*^ is itself a *simplicial complex*. We are interested in tracking the birth and death of homology generators along filtration.

Given a *simplicial complex* *K* with its filtration, the *p-persistent kth homology group* of *K*^*i*^ is defined as $H_{k}^{p}\left(K^{i}\right)=Z_{k}\left(K^{i}\right)/\left(B_{k}\left(K^{i+p}\right)\cap Z_{k}\left(K^{i}\right)\right)$. Intuitively, this records the homology classes of *K*^*i*^ that are persistent at least until *K*^*i*+*p*^. Persistent homology allows us to not only compute *n*-dimensional holes at a specific setup, but also compute the parameter values corresponding to the birth and death of the *n*-dimensional holes along the filtration.

A generator (equivalence class) in *H*_*k*_(*K*^*i*^) which does not exist in *H*_*k*_(*K*^*i*−1^) or *H*_*k*_(*K*^*j*^) and lasts until *H*_*k*_(*K*^*j*−1^), under the mappings along the sequence of homology groups induced by inclusion maps along the sequence of simplicial complexes, is associated with the interval [*x*_*i*_, *x*_*j*_), where *x*_*i*_ and *x*_*j*_ are the filtration levels associated to *K*^*i*^ and *K*^*j*^. A collection of these intervals tracks the appearing and disappearing of homology generators along the filtration process. Such collections of intervals can be visualized by stacking horizontal line segments (barcodes) or by plotting in a plane (persistence diagrams). The collection of intervals associated to the *k*th homology group is called the *k*th dimensional barcodes.

#### Simplicial complexes and filtration

Given a finite set of points *X* and a non-negative scale parameter *r*, the Vietoris-Rips complex and alpha complex are constructed as follows.

With a predefined distance function *d*(⋅, ⋅) in *X*, a subset *X*′ of *X* forms a simplex if *d*(*x*_*i*_, *x*_*j*_) ≤ *r* for all *x*_*i*_, *x*_*j*_ ∈ *X*′. The collection of all such simplices is the *Vietoris-Rips* complex of the finite metric space *X* with scale parameter *r* denoted by *Rips*(*X*, *r*). It is obvious that *Rips*(*X*, *r*) ⊆ *Rips*(*X*, *r*′) for *r* ≤ *r*′.

With *Alpha*(*X*, *r*) being the *alpha complex* of *X* with the scale parameter *r* and given the Delaunay triangulation induced by the Voronoi diagram of *X*, a simplex in the Delaunay triangulation belongs to *Alpha*(*X*, *r*) if all its *1-faces* (*1-simplex* as subset of the simplex) have length no greater than 2*r*. Similar to Rips complex, alpha complex also has the property that *Alpha*(*X*, *r*) ⊆ *Alpha*(*X*, *r*′) for *r* ≤ *r*′.

### Biological considerations

The development of persistent homology was motivated by its potential in the dimensionality reduction, abstraction and simplification of biomolcular complexity [36]. In the early applications of persistent homology to biomolecules, emphasis was given on major or global features (long-persistent features) to derive descriptive tools. For example, persistent homology was used to identify the tunnel in a Gramicidin A channel [36] and to study membrane fusion [118]. For the predictive modeling of biomolecules, features of a wide range of scales might all be important to the target quantity [26]. At the global scale, the biomolecular conformation should be captured. At the intermediate scale, the smaller intra-domain cavities need to be identified. At the most local scale, the important substructures should be addressed, such as the pyrrolidine in the side chain of proline. These features of different scales can be reflected by barcodes with different centers and persistences. Therefore, applications in biomolecules can make a more exhaustive use of persistent homology [26, 87], compared to some other applications where only global features matter while most local features are mapped to noise. Earlier use of persistent homology was focused on qualitative analysis. Only recently had persistent homology been devised as a quantitative tool [26, 87]. While the aforementioned applications are descriptive and regression based analysis, we have also applied persistent homology to predictive modeling of biomolecules [92]. However, biomolecules are both structurally and biologically complex. Their geometric and biological complexities include covalent bonds, non-covalent interactions, effects of chirality, cis and trans distinctions, multi-leveled protein structures, and protein-ligand and protein-nucleic acid complexes. Covering a large range of spatial scales is not enough for a powerful model. The biological details should also be explored. We address the underlying biology and physics by modifying the distance function and selecting various sets of atoms according to element types, to describe different interactions. Some biological considerations are discussed in this section.

#### Covalent bonds

Covalent bonds are formed via shared electron pairs or bonding pairs. The lengths and the number of covalent bonds can be easily detected from 0th dimensional barcodes. For macromolecules, the same type of covalent bonds have very similar bond lengths and thus 0th dimensional barcode patterns.

#### Non-covalent interactions

Non-covalent interactions play a critical role in maintaining the 3D structure of biomolecules and mediating chemical and biological processes, such as solvation, binding, protein-DNA specification, molecular self-assembly, etc. Physically, non-covalent interactions are due to electrostatic, van der Waals forces, hydrogen bonds, *π*-effects, hydrophobic effects, etc. The ability to characterize non-covalent interactions is an essential task in any methodological development. The 1st and 2nd dimensional barcodes are suitable for the characterization of the arrangement of such interactions in a larger scale. Additionally, we propose multi-level persistence and electrostatic persistence to reveal local and pairwise non-covalent interactions via 0th dimensional barcodes as well.

#### Chirality, cis effect and trans effect

Chirality, cis and trans effects are geometric properties of many molecules. Among them, chirality is a symmetry property such that a chiral molecule cannot be superposed on its mirror image. Cis and trans effects are due to molecular steric and electronic effects. Chirality, cis and trans effects often play a role in molecular kinetics, activity and catalysis, and thus their characterization is an important issue in developing topological methods. These effects should be reflected from barcodes of various dimensions.

#### Multi-leveled protein structures

Protein structures are typically described in terms of primary, secondary, tertiary and quaternary levels. The protein primary structure is the linear sequence of amino acids in the polypeptide chain. Protein secondary structure refers to the local 3D structure of protein segments containing mainly *α*-helix and *β*-sheets, which are highly regular and can be easily detected by distinct Frenet-Serret frames. A tertiary structure refers to the 3D structure of a single polypeptide chain. Its formation involves various non-covalent and covalent interactions including salt bridges, hydrophobic effects, and often disulfide bonds. A quaternary structure refers to the aggregation of two or more individual folded protein subunits into a 3D multi-subunit complex. Protein structures are further complicated by its functional domains, motifs, and particular folds. The protein structural diversity and complexity result in the challenge and opportunity for methodological developments. Various persistent homology techniques, including multi-component, multi-level, multi-dimensional [119], multi-resolution [90], electrostatic, and interactive [27] persistent homologies have been designed either in our earlier work or in this paper for protein structural diversity and complexity.

#### Protein-ligand, protein-protein, and protein-nucleic acid complexes

Topological characterization of proteins is further complicated by protein interactions or binding with ligands (drugs), other proteins, DNA and/or RNA molecules. Although a normal protein involves only carbon (C), hydrogen (H), nitrogen (N), oxygen (O) and sulfur (S) atoms, its protein-ligand complexes bring a variety of other elements into the play, including, phosphorus (P), fluorine (F), chlorine (Cl), Bromine (Br), iodine (I), and many important biometals, such as calcium (Ca), potassium (K) sodium (Na), iron (Fe), copper (Cu), cobalt (Co), zinc (Zn), manganese (Mn), chromium (Cr), vanadium (V), tin (Sn), and molybdenum (Mo). Each biological element has important biological functions and its presence in biomolecules should be treated uniformly as a set of points in the point cloud data. The interaction of protein and nucleic acids can be very intricate. Qualitatively, multiscale and multi-resolution persistent homology demonstrates interesting features in 3D DNA structures [89]. Typically, 3D RNA structures are more flexible and difficult to extract topological patterns. Interactive persistent homology, element specific persistent homology and binned representation for persistent homology outputs were designed to deal with interactions between protein-ligand, protein-protein, and protein-nucleic acid complexes [14, 27, 94]. These approaches worked well in protein-mutation site interactions [14]. Additionally, multi-level persistent homology and electrostatic persistence proposed in this work are useful tools to describe some other specific interactions.

### Element specific persistent homology

One important issue is how to protect chemical and biological information during the topological simplification. As mentioned earlier, one should not treat different types of atoms as homogeneous points in a point cloud data. To this end, element specific persistent homology or multi-component persistent homology has been proposed to retain biological information in topological analysis [14, 27, 94]. The element selection is similar to a predefined vertex color configuration for graphs.

When all atoms are passed to persistent homology algorithms, the information extracted mainly reflects the overall geometric arrangement of a biomoelcule at different spatial scales. By passing only atoms of certain element types or of certain roles to the persistent homology analysis, different types of interactions or geometric arrangements can be revealed. In protein-ligand binding modeling, the selection of all carbon atoms characterizes the hydrophobic interaction network whilst the selection of all nitrogen and/or oxygen atoms characterizes hydrophilic network and the network of potential hydrogen bonds. In the protein structural analysis, computation on all atoms can identify geometric voids inside the protein which may suggest structural instability and computation on only C_*α*_ atoms reveals the overall structure of amino acid backbones. In addition, combination of various selections of atoms based on element types provides very detailed description of the biomolecular system and the hidden relationships from the structure to function can then be learned by machine learning algorithms. This may lead to the discovery of important interactions not realized as *a prior*. This can be realized by passing the set of atoms of the selected element types to the persistent homology computation. This concept is used with the various definitions of distance matrix discussed as follows.

### Distance matrix induced persistent homology

Biomolecular systems are not only complex in geometry, but also in chemistry and biology. To effectively describe complex biomolecular systems, it is necessary to modify the filtration process. There are three commonly used filtrations, namely, radius filtration, distance matrix filtration, and density filtration, for biomolecules [26, 90]. A distance matrix defined with smoothed cutoff functions was proposed in our earlier work to deal with interactions within a spatial scale of interest in biomolecules [26]. In the present work, we introduce more distance matrices to enhance the representational power of persistent homology and to cover some important interactions that were not covered in our earlier works. The distance matrices can be used with a more abstract construction of simplicial complexes, such as Vietoris-Rips complex.

#### Multi-level persistent homology

Small molecules such as ligands in protein-ligand complexes usually contain fewer atoms than large biomolecules such as proteins. Bonded atoms stay closer than non-bonded ones in most cases. As a result, the collection of 0th dimensional bars will mostly provide the information about the length of covalent bonds and the higher dimension barcodes will most likely be very sparse. It is difficult to capture non covalent bond interactions among atoms especially hydrogen bonds and van der Waals pairwise interactions in 0th dimensional barcodes. In order to describe non covalent interactions, we propose multi-level persistent homology, by simply modifying the distance matrix, similar to the idea of modifying distance matrix to emphasize on the interactions between protein and ligand [27]. Given the original distance matrix **M** = (*d*_*ij*_) with 1 ≤ *i*, *j* ≤ *N*, the modified distance matrix is defined as

$$\begin{array}{c} {\tilde{M}}_{ij}=\{\begin{array}{c} d_{\infty },\;\text{if}\;\text{atoms}\;i\;\text{and}\;j\;\text{are}\;\text{bonded},\\ M_{ij},\;\text{otherwise}, \end{array} \end{array}$$ (3)

where *d*_∞_ is a large number which is set to be greater than the upper limit of the filtration value chosen by a persistent homology algorithm. Note that this matrix may fail to satisfy triangle inequality whilst still satisfies the construction principle of Rips complex.

**Fig 8:**
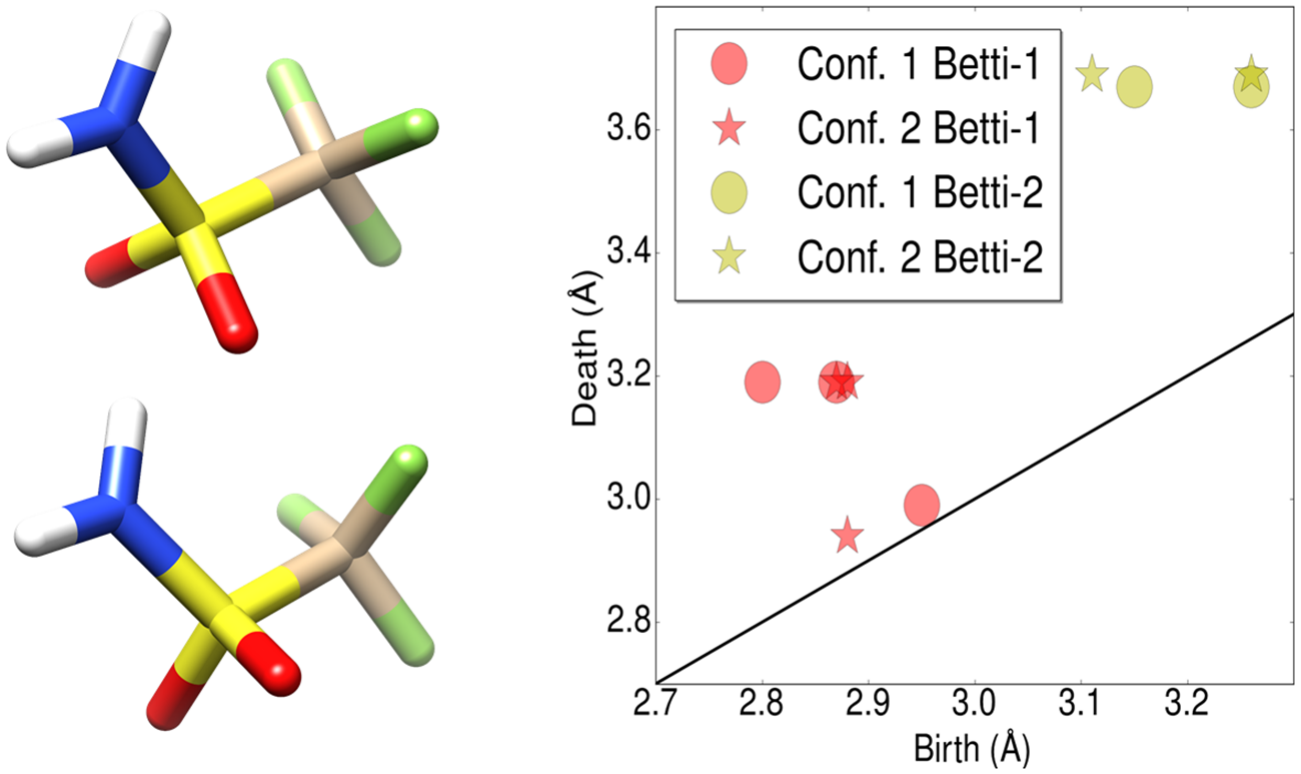
Multi-level persistent homology on simple small molecules. Illustration of representation ability of $\tilde{M}$ in reflecting structural perturbations among conformations of the same molecule. Left: The structural alignment of two conformations of the ligand in protein-ligand complex (PDB:1BCD). Right: The persistence diagram showing the 1st and 2nd dimensional results generated using Rips complex with $\tilde{M}$ for two conformations. It is worth noticing that the barcodes generated using Rips complex with **M** are identical for the two conformations.

The present multi-level persistent homology is able to describe any selected interactions of interest and delivers two benefits in characterizing biomolecules. Firstly, the pairwise non-covalent interactions can be reflected by the 0th dimensional barcodes. Secondly, such treatment generates more higher dimensional barcodes and the small structural fluctuation among different conformations of the same molecule can be captured. The persistent barcode representation of the molecule can be significantly enriched to better distinguish between different molecular structures and isomers. As an illustration, we take the ligand from the protein-ligand complex with PDB code “1BCD” which only has 10 atoms. A different conformation of the ligand is generated by using the Frog2 web server [120]. The persistent barcodes generated using Rips complex with the distance matrices **M** are identical and only have 0th dimensional bars due to the simple structure. In this case, the 0th dimensional bars only reflect the length of each bond and therefore fail to distinguish the two slightly different conformations of the same molecule. However, when the modified distance matrices $\tilde{M}$ are employed, the barcode representation is significantly enriched and is able to capture the tiny structural perturbation between the conformations. An illustration of the outcome from the modified distance matrix $\tilde{M}$ is shown in Fig 8. A general *n*th level persistence characterization of molecules can be obtained with the distance matrix ${\tilde{M}}^{n}$ as,

$$\begin{array}{c} {\tilde{M}}_{ij}^{n}=\{\begin{array}{c} d_{\infty },D\left(i,j\right)\leq n\\ M_{ij},\;\text{otherwise}, \end{array} \end{array}$$ (4)

where *D*(*i*, *j*) is the smallest number of bonds to travel from atom *i* to atom *j* and *d*_∞_ is some number greater than the upper limit of the filtration value.

#### Interactive persistent homology

In protein-ligand binding analysis and analysis involving interactions, we are interested in the change of topological invariants induced by interactions that are caused by binding or other processes. Similar to the idea of multi-level persistent homology, we can design a distance matrix to focus on the interactions of interest. For a set of atoms, *A* = *A*_1_ ∪ *A*_2_ with *A*_1_ ∩ *A*_2_ = ∅ where only interactions between atoms from *A*_1_ and atoms from *A*_2_ are of interest [27]. The interactive distance matrix $\hat{M}$ is defined as

$$\begin{array}{c} {\hat{M}}_{ij}=\{\begin{array}{c} M_{ij},\;\text{if}\;a_{i}\in A_{1},\;a_{j}\in A_{2}\;\text{or}\;a_{i}\in A_{2},\;a_{j}\in A_{1},\\ d_{\infty },\;\text{otherwise}, \end{array} \end{array}$$ (5)

where **M** is the original distance matrix induced from Euclidean metrics or other correlation function based distances, *a*_*i*_ and *a*_*j*_ are atoms *i* and *j*, and *d*_∞_ is a number greater than the upper limit of the filtration value. In applications, *A*_1_ and *A*_2_ can be respectively a set of atoms of the protein and a set of atoms of the ligand in a protein-ligand complex. In this case, the characterization of interactions between ligand and protein is an important task. In the modeling of point mutation induced protein stability changes, *A*_1_ could be the set of atoms at the mutation site and *A*_2_ could be the set of atoms of surrounding residues close to the mutation site. Similar treatment can be used for protein-protein and protein-nucleic acid interactions.

#### Correlation function based persistent homology

For biomolecules, the interaction strength between pair of atoms usually does not align linearly to their Euclidean distances. For example, van der Waals interaction is often described by the Lennard-Jones potential. Therefore, kernel function filtration can be used to emphasize certain geometric scales. Correlation function based filtration matrix was introduced in our earlier work [26]:

$$\begin{array}{c} {\bar{M}}_{ij}=1-\Phi \left(d_{ij},\eta_{ij}\right), \end{array}$$ (6)

where Φ(*d*_*ij*_, *η*_*ij*_) is a radial basis function and *η*_*ij*_ is a scale parameter. This filtration can be incorporated in the element specific persistent homology

$$\begin{array}{c} {\overset{`}{M}}_{ij}=\{\begin{array}{c} d_{\infty },\;\text{if}\;\text{atom}\;i\;\text{or}\;\text{atom}\;j\;\in U,\\ 1-\Phi (d_{ij},\eta_{ij}),\;\text{otherwise}. \end{array} \end{array}$$ (7)

Additionally, one can simultaneously use two or more correlation functions characterized by different scales to generate a multiscale representation of biomolecules [106].

#### Flexibility and rigidity index based filtration matrix

One form of the correlation function based filtration matrix is constructed by flexibility and rigidity index. In this case, the Lorentz function is used in Eq (7)

$$\begin{array}{c} \Phi \left(d_{ij};\eta_{ij},\nu \right)=\frac{1}{1+{\left(\frac{d_{ij}}{\eta_{ij}}\right)}^{\nu }}, \end{array}$$ (8)

where *d*_*ij*_ is the Euclidean distance between point *i* and point *j* and *η*_*ij*_ is a parameter controlling the scale and is related to radius of two atoms. When distance matrices based on such correlation functions are used, patterns at different spatial scales can be addressed separately by altering the scale parameter *η*_*ij*_. Note that the rigidity index is given by [121]

$$\begin{array}{c} \mu_{i}=\sum_{j}\Phi \left(d_{ij};\eta_{ij},\nu \right). \end{array}$$ (9)

This expression is closely related to the rigidity density based volumetric filtration [90].

#### Electrostatic persistence

Electrostatic effects are some of the most important effects in biomolecular structure, function, and dynamics. The embedding of electrostatics in topological invariants is of particular interest and can be very useful in describing highly charged biomolecules such as nucleic acids and their complexes. We introduce electrostatics interaction induced distance functions in Eq (10) to address the electrostatic interactions among charged atoms. The abstract distance between two charged particles are rescaled according to their charges and their geometric distance, and is modeled as

$$\begin{array}{c} \Phi \left(d_{ij},q_{i},q_{j};c\right)=\frac{1}{1+\exp(-cq_{i}q_{j}/d_{ij})}, \end{array}$$ (10)

where *d*_*ij*_ is the distance between the two atoms, *q*_*i*_ and *q*_*j*_ are the partial charges of the two atoms, and *c* is a nonzero tunable parameter. *c* is set to a positive number if opposite-sign charge interactions are to be addressed and is set to a negative number if same-sign charge interactions are of interest. The form of the function is adopted from sigmoid function which is widely used as an activation function in artificial neural networks. Such function regularizes the input signal to the [0, 1] interval. Other functions can be similarly used. This formulation can be extended to systems with dipole or higher order multipole approximations to electron density. The weak interactions due to long distances or neutral charges result in correlation values close to 0.5. When *c* > 0, the repulsive interaction and attractive interaction deliver the correlation values in (0.5, 1) and (0, 0.5) respectively. The distances induced by Φ(*d*_*ij*_, *q*_*i*_, *q*_*j*_; *c*) are used to characterize electrostatic effects. The parameter *c* is rather physical but chosen to effectively spread the computed values over the (0, 1) interval so that the results can be used by machine learning methods. Another simple choice of charge correlation functions is

$$\begin{array}{c} \Phi \left(d_{ij},\eta_{ij},q_{i},q_{j}\right)=q_{i}q_{j}\exp\left(-d_{ij}/\eta_{ij}\right). \end{array}$$

However, this choice will lead to a different filtration domain. Additionally, a charge density can be constructed

$$\begin{array}{c} \mu^{c}\left(r\right)=\sum_{j}q_{j}\exp(-∥r-r_{j}∥/\eta_{j}), \end{array}$$ (11)

where **r** is a position vector, ∥**r** − **r** *_j_*∥ is the Euclidean distance between **r** and *j*th atom position **r** *_j_* and *η*_*j*_ is a scale parameter. Eq (11) can be used for electrostatic filtration as well. In this case, the filtration parameter can be the charge density value and cubical complex based filtration can be used.

#### Multi-component persistent homology

Multicomponent persistent homology refers to the construction of multiple persistent homology components from a given object to describe its properties. Obviously, element specific persistent homology leads to multi-component persistent homology. Nevertheless, in element specific persistent homology, the emphasis is given to the appropriate selection of important elements for describing certain biological properties or functions. For example, in biological context, electronegative atoms are selected for describing hydrogen bond interactions, polar atoms are selected for describing hydrophilic interactions, and carbon atoms are selected for describing hydrophobic interactions. Note that in chemical context, an atom may have many sharply different chemical and physical properties, depending on its oxidation states. Whereas, in multicomponent persistent homology, the emphasis is placed on the systematic generation of topological invariants from different combinatorial possibilities and the construction of 2D or high-dimensional persistent maps for deep convolutional neural networks.

### Feature generation from topological invariants

Barcode representation of topological invariants offers a visualization of persistent homology analysis. In machine learning analysis, we convert the barcode representation of topological invariants into structured feature arrays for machine learning. To this end, we introduce two methods, i.e., counts in bins, barcode statistics, and persistence diagram slice and statistics, to generate feature vectors from sets of barcodes. These methods are discussed below. Python code is given in S1 Code for the generation of features used in the final models in the Results section.

#### Counts in bins

For a given set of atoms *A*, we denote its barcodes as **B** = {*I*_*α*_}_*α* ∈ *A*_ and represent each bar by an interval *I*_*α*_ = [*b*_*α*_, *d*_*α*_], where *b*_*α*_ and *d*_*α*_ are respectively the birth and death positions on the filtration axis. The length of each bar, or the persistence of topological invariant is given by *p*_*α*_ = *d*_*α*_ − *b*_*α*_. To locate the position of all bars and persistences, we further split the set of barcodes on the filtration axis into a predefined collection of *N* bins $\text{Bin}={\left\{{\text{Bin}}_{i}\right\}}_{i=1}^{N}$ with Bin_*i*_ = [*l_i_*, *r_i_*], where *l*_*i*_ and *r*_*i*_ are the left and the right positions of the *i*th bin. We generate features by counting the numbers of births, deaths, and persistences in each bin, which leads to three counting feature vectors, namely, counts of birth $F_{b}^{C}$, death $F_{d}^{C}$, and persistence $F_{p}^{C}$,

$$\begin{array}{c} \begin{array}{cc} F_{b,i}^{C}\left(B\right) & =∥\{\left[b_{\alpha },d_{\alpha }\right]\in B|l_{i}\leq b_{\alpha }\leq r_{i}\}∥,1\leq i\leq N,\\ F_{d,i}^{C}\left(B\right) & =∥\{\left[b_{\alpha },d_{\alpha }\right]\in B|l_{i}\leq d_{\alpha }\leq r_{i}\}∥,1\leq i\leq N,\\ F_{p,i}^{C}\left(B\right) & =∥\{\left[b_{\alpha },d_{\alpha }\right]\in B|b_{\alpha }\leq r_{i}\;\text{or}\;l_{i}\leq d_{\alpha }\}∥,1\leq i\leq N, \end{array} \end{array}$$ (12)

where ‖ ⋅ ‖ is the cardinality of a set. Note that the above discussion should be applied to three topological dimensions, i.e., barcodes of the 0th dimension (**B**^**0**^), 1st dimension (**B**^**1**^) and 2nd dimension (**B**^**2**^). In general, this approach enables the description of bond lengths, including the length of non-covalent interactions, in biomolecules and was referred to as binned persistent homology in our earlier work [14, 27, 94].

#### Barcode statistics

Another method of feature vector generation from a set of barcodes is to extract important statistics of barcode collections such as maximum values and standard deviations. Given a set of bars **B** = {[*b*_*α*_, *d*_*α*_]}_*α*∈*A*_, we define sets of **Birth** = {*b*_*α*_}_*α*∈*A*_, **Death** = {*d*_*α*_}_*α*∈*A*_, and **Persistence** = {*d*_*α*_ − *b*_*α*_}_*α*∈*A*_. Three statistic feature vectors $F_{b}^{S}$, $F_{d}^{S}$, and $F_{p}^{S}$ can then be generated in the sense of the statistics of the collection of barcodes. For example, $F_{b}^{S}$ consists of avg(**Birth**), std(**Birth**), max(**Birth**), min(**Birth**), sum(**Birth**), and cnt(**Birth**), where avg(⋅) is the average value of a set of numbers, std(⋅) is the standard deviation of a set of numbers, max(⋅) and min(⋅) are maximum and minimum values in a set of numbers, sum(⋅) is the summation of elements in a set of numbers, and cnt(⋅) is the count of elements in a set. The generation of $F_{d}^{S}$ is the same by examining the set **Death**. $F_{p}^{S}$ contains the same information with two extra terms, the birth and death values of the longest bar. Statistics feature vectors are collected from barcodes of three topological dimensions, i.e., the 0th, 1st, and 2nd dimensions.

#### Persistence diagram slice and statistics

**Fig 9:**
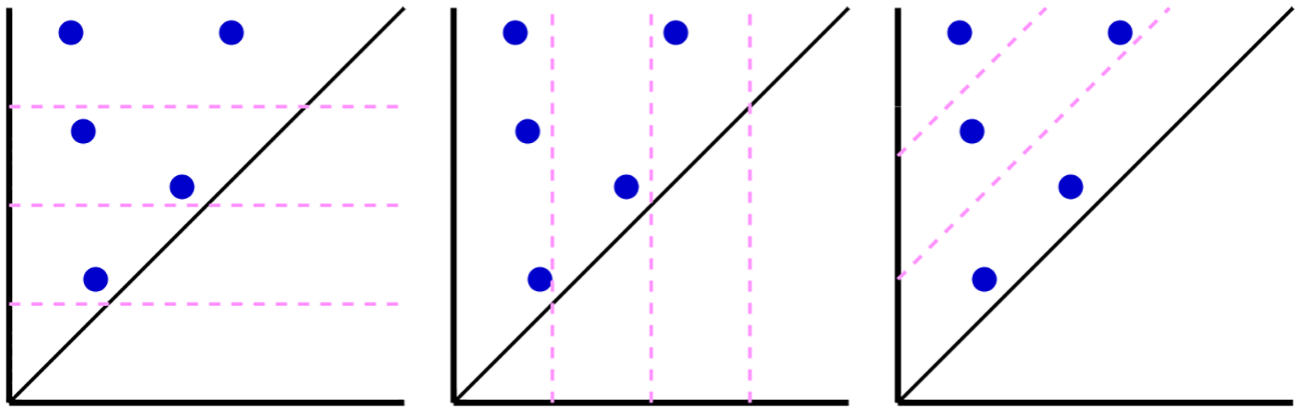
Illustration of dividing set of barcodes into subsets. The barcodes are plotted as persistence diagrams with the horizontal axis being birth and the vertical axis being death. From left to right, the subsets are generated according to the slicing of death, birth, and persistence values.

A more thorough description of sets of barcodes is to first divide the sets into subsets and extract features analogously to the *barcode statistics* method. As shown in Fig 9, a persistence diagram can be divided into slices in different directions. The barcodes that fall in each slice form a subset. Each subset is described in terms of feature vector by using the *barcode statistics* method. When the persistence diagram is sliced horizontally, members in each subset have similar death values and the barcode statistics feature vector is generated for the set of birth values. Similarly, members in each subset have similar birth values if the persistence diagram is sliced vertically, and the barcode statistics feature vector is generated for the set of death values. The barcode statistics feature vectors are generate for both set of birth values and set of death values if the persistence diagram is sliced diagonally, where members in each subset have similar persistence. This type of feature vector generation describes the set of barcodes in more detail but will produce longer feature vectors.

#### 2D representation

The construction of multi-dimensional persistence is an interesting topic in persistent homology. In general, it is believed that multi-dimensional persistence has better representational power for complex systems described by multiple parameters [43]. Although multidimensional persistence is hard to compute, one can compute persistence for one parameter while fixing the rest of the parameters to a sequence of fixed values. In the case where there are two parameters, a bifiltration can be done by taking turns to fix one parameter to a sequence of fixed values while computing persistence for the other parameter. For example, one can take a sequence of resolutions and compute persistence for distance with each fixed resolution. The sequence of outputs can be stacked to form a multidimensional representation [119].

Computing persistence multiple times and stacking the results is especially useful when the parameters that are not chosen to be the filtration parameter are naturally discrete with underlying orders. For example, the multi-component or element specific persistent homology will result in many persistent homology computations over different selections of atoms. These results can be ordered by the percentage of atoms used of the whole molecule or by their importance scores in classical machine learning methods. Also, multiple underlying dimensions exist in the element specific persistent homology characterization of molecules. This property enables 2D or 3D topological representation of molecules. Based on the observation that the performance of the predictor degenerates when too many element combinations are used, we order the element combinations according to their individual performance on the task using methods of ensemble of trees. Combining the dimension of spatial scale and dimension of element combinations, a 2D topological representation is obtained. Such representation is expected to work better in the case of complex geometry such as protein-ligand complexes. With $E={\left\{E_{j}\right\}}_{j=1}^{N_{E}}$ denoting the collection of element combinations ordered by their individual importance scores on the task and **B**^*k*^(*E*_*i*_) being the *k*th dimensional barcodes obtained with atoms of element combination *E*_*j*_, eight 2D representations are defined as

$$\begin{array}{l} \{F_{d,i}^{C}\left(B^{0}\left(E_{j}\right)\right),F_{p,i}^{C}\left(B^{0}\left(E_{j}\right)\right),F_{b,i}^{C}\left(B^{1}\left(E_{j}\right)\right),F_{d,i}^{C}\left(B^{1}\left(E_{j}\right)\right),\\ {F_{p,i}^{C}\left(B^{1}\left(E_{j}\right)\right),F_{b,i}^{C}\left(B^{2}\left(E_{j}\right)\right),F_{d,i}^{C}\left(B^{2}\left(E_{j}\right)\right),F_{p,i}^{C}\left(B^{2}\left(E_{j}\right)\right)\}}_{i=1,\cdots ,N}^{j=1,\cdots ,N_{E}}, \end{array}$$ (13)

where $F_{\gamma ,i}^{C}$ with *γ* = *b*, *d*, *p* is the barcode counting rule defined in Eq (13). For 0th dimensional, since all bars start from zero, there is no need for $F_{b,i}^{C}\left(B^{0}\left(E_{j}\right)\right)$. These eight 2D representations are regarded as eight channels of a 2D topological image. In protein-ligand binding analysis, 2D topological features are generated for the barcodes of a protein-ligand complex and for the differences between barcodes of the protein-ligand complex and those of the protein. Therefore, we have a total of 16 channels in a 2D image for the protein-ligand complex. This 16-channel image can be fed into the training or the prediction of convolutional neural networks.

**Fig 10:**
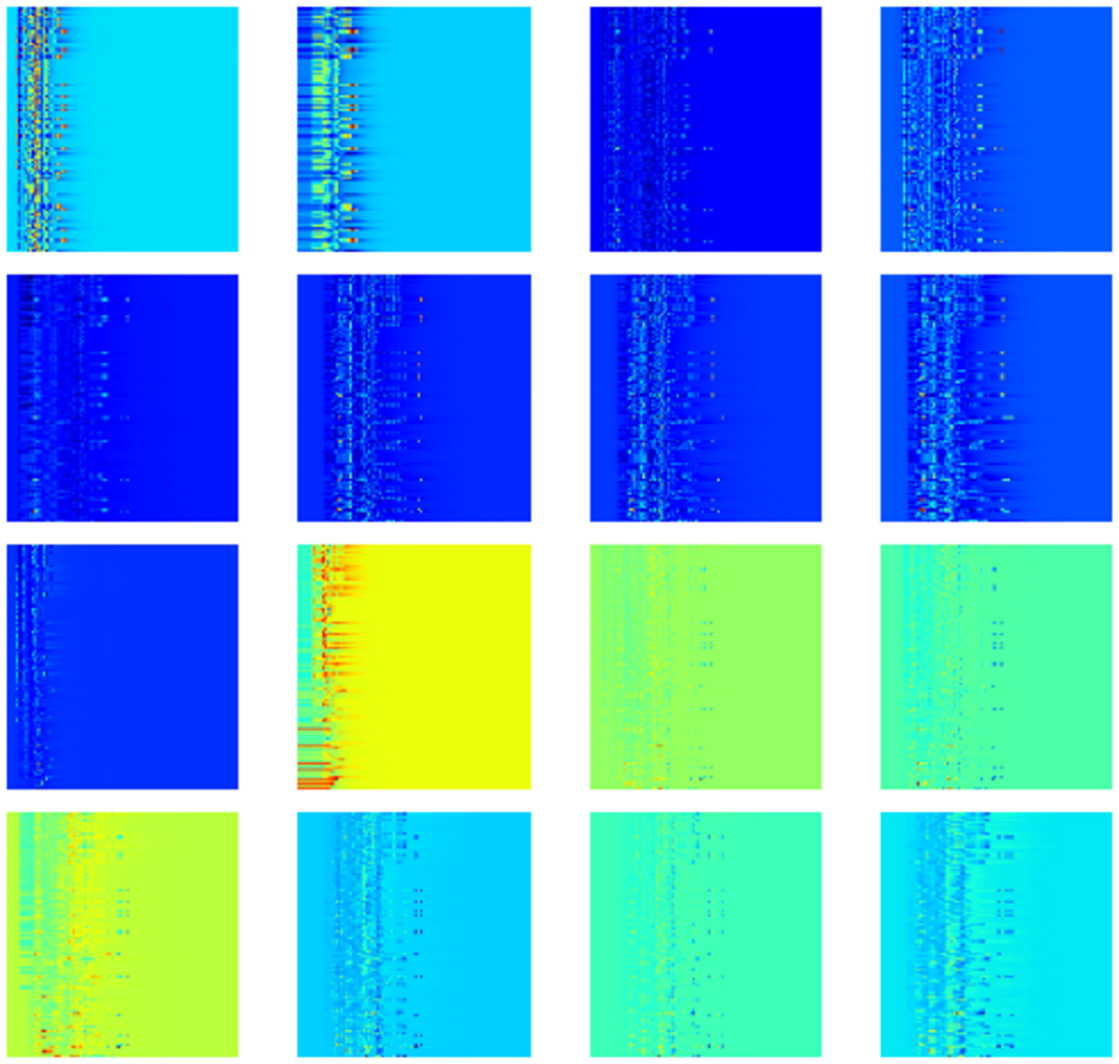
The 2D topological maps of the 16 channels of sample 1wkm. The top 8 maps are for protein-ligand complex and the other 8 maps are for the difference between protein-ligand complex and protein only. For each map, the horizontal axis is the dimension of spatial scale and the vertical axis is element combinations ordered by their importance.

In the characterization of protein-ligand complexes using alpha complexes, 2D features are generated from the alpha complex based on persistent homology computations of protein and protein-ligand complex. A total of 128 element combinations are considered. The [0, 12]Å interval is divided into 120 equal length bins, which defines the resolution of topological images. Therefore, the input feature for each sample is a 120×128×16 tensor. Fig 10 illustrates 16 channels of sample 1wkm in PDBBind database. These images are directly used in deep convolutional neural networks for training and prediction.

When there are fewer element combinations considered which can hardly form another axis, the axis of element combinations can be added into the original channels to form 1D representations that can be used in 1D CNN.

### Machine learning algorithms

Three machine learning algorithms, including k-nearest neighbors (KNN) regression, gradient boosting trees and deep convolutional neural networks, are integrated with our topological representations to construct topological learning algorithms.

#### K-nearest neighbors algorithm via barcode space metrics

One of the simplest machine learning algorithms is k-nearest neighbors (KNN) for classification or for regression. In KNN regression, for a given object, its property values is obtained by the average or the weighted average of the values of its *k* nearest neighbors induced by a given metric of similarity. Then, the problem becomes how to construct a metric on the dataset.

In the present work, instead of computing similarities from constructed feature vectors, the similarity between biomolecules can simply be derived from distances between barcodes generated from different biomolecules. Popular barcode space metrics include the bottleneck distance [122] and more generally, the Wasserstein metrics [95, 96]. The definition of the two metrics is summarized as follows.

Given two bars *I*_1_ = [*b*_1_, *d*_1_] and *I*_2_ = [*b*_2_, *d*_2_] regarded as ordered pairs in $R^{2}$, the *l*^∞^ distance between the two bars is defined as Δ(*I*_1_, *I*_2_) = max(|*b*_2_ − *b*_1_|, |*d*_2_ − *d*_1_|). For a single bar *I* = [*b*, *d*], λ(*I*) is defined as λ(*I*) = (*d* − *b*)/2 which helps reflect the difference between the existence of the bar itself and the void. For two finite barcodes $B_{1}={\left\{I_{\alpha }^{1}\right\}}_{\alpha \in A}$ and $B_{2}={\left\{I_{\beta }^{2}\right\}}_{\beta \in B}$ and a bijection *θ* from *A*′ ⊆ *A* to *B*′ ⊆ *B*, the penalty of *θ* is defined as

$$\begin{array}{c} P\left(\theta \right)=\max(\max_{\alpha \in A^{\prime }}\left(\Delta \left(I_{\alpha }^{1},I_{\theta (\alpha )}^{2}\right)\right),\max_{\alpha \in A-A^{\prime }}\left(\lambda \left(I_{\alpha }^{1}\right)\right),\max_{\beta \in B-B^{\prime }}\left(\lambda \left(I_{\beta }^{2}\right)\right)). \end{array}$$ (14)

Intuitively, a bijection *θ* is penalized for linking two bars with large difference and for ignoring long bars from either set. The bottleneck distance is defined as $d^{\infty }({\text{B}}_{1},{\text{B}}_{2})=\underset{\theta }{\min}\;P(\theta )$, where the minimum is taken over all possible bijections from subsets of *A* to subsets of *B*.

The Wasserstein metric, a *L*_*p*_ generalized analog to the bottleneck distance can be defined with the penalty [96]

$$\begin{array}{c} P^{p}\left(\theta \right)=\sum_{\alpha \in A^{\prime }}\Delta {\left(I_{\alpha }^{1},I_{\theta (\alpha )}^{2}\right)}^{p}+\sum_{\alpha \in A-A^{\prime }}\lambda {\left(I_{\alpha }^{1}\right)}^{p}+\sum_{\beta \in B-B^{\prime }}\lambda {\left(I_{\beta }^{2}\right)}^{p} \end{array}$$ (15)

and the corresponding distance $d^{p}\left(B_{1},B_{2}\right)={\left(\min_{\theta }P^{p}\left(\theta \right)\right)}^{\frac{1}{p}}$. It approaches the bottleneck distance by setting *p* goes to infinity. In this work, we choose *p* = 2.

Wasserstein metric measures the closeness of barcodes generated from different biomolecules. It will be interesting to consider other distances for metric spaces, such as Hausdorff distance, Gromov-Hausdorff distance [123], and Yau-Hausdorff distance [124] for biomolecular analysis. However, an exhaustive study of this issue is beyond the scope of the present work.

The barcode space metrics can be directly used to assess the representation power of various persistent homology methods on biomolecules without being affected by the choice of machine learning models and hyperparameters. We show in the section of results that the barcode space metrics induced similarity measurement is significantly correlated to molecule functions.

Wasserstein metric measures from biomolecules can also be directly implemented in a kernel based method such as nonlinear support vector machine algorithm for classification and regression tasks. However, this aspect is not explored in the present work.

#### Gradient boosting trees

Gradient boosting trees is an ensemble method which ensembles individual decision trees to achieve the capability of learning complex feature target maps and can effectively prevent overfitting by using shrinkage technique. The gradient boosting trees method is realized using the GradientBoostingRegressor module in scikit-learn software package [114] (version 0.17.1). A set of parameters found to be efficient in our previous study on the protein-ligand binding affinity prediction [27] is used uniformly unless specified. The parameters used are *n_estimators = 20000, max_depth = 8, learning_rate = 0.005, loss = ‘ls’, subsample = 0.7, max_features = ‘sqrt’*.

#### Deep convolutional neural networks

The deep convolutional neural networks in this work are implemented using Keras [125] (version 1.1.2) with Theano backend [126] (version 0.8.2).

**Fig 11:**
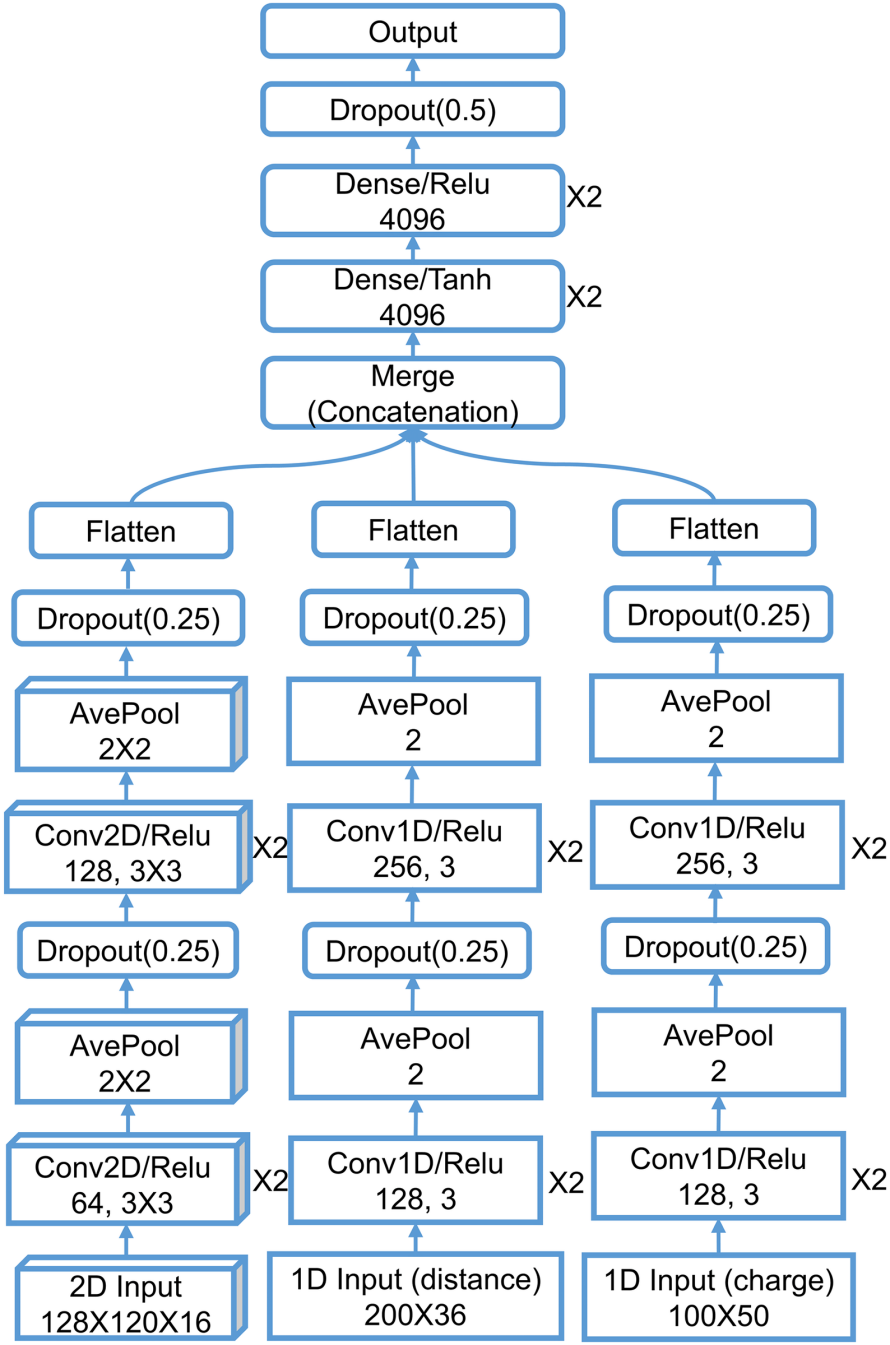
The network architecture of TopBP-DL. The structured layers are shown in boxes/rectangles with sharp corners for 2D/1D image-like content and the unstructured layers are shown in rectangles. The numbers in convolution layers mean the number of filters and filter size from left to right. The dense layers are drawn with number of neurons and activation function. The pooling size of the pooling layers and dropout rate of the dropout layers are listed. The layers that are repeated *n* times are marked with “×*n*” sign on the right side of the layer.

For TopBP-DL(Complex), a widely used convolutional neural network architecture is employed beginning with convolution layers followed by dense layers. Due to the limited computation resources, parameter optimization is not performed, while most parameters are adopted from our earlier work [94]. Reasonable parameters are assigned manually. The detailed architecture is shown in Fig 11. The Adam optimizer with learning rate 0.0001 is used. The loss function is the mean squared error function. The network is trained with a batch size of 16 and 150 epochs. The training data is shuffled for each epoch.

**Fig 12:**
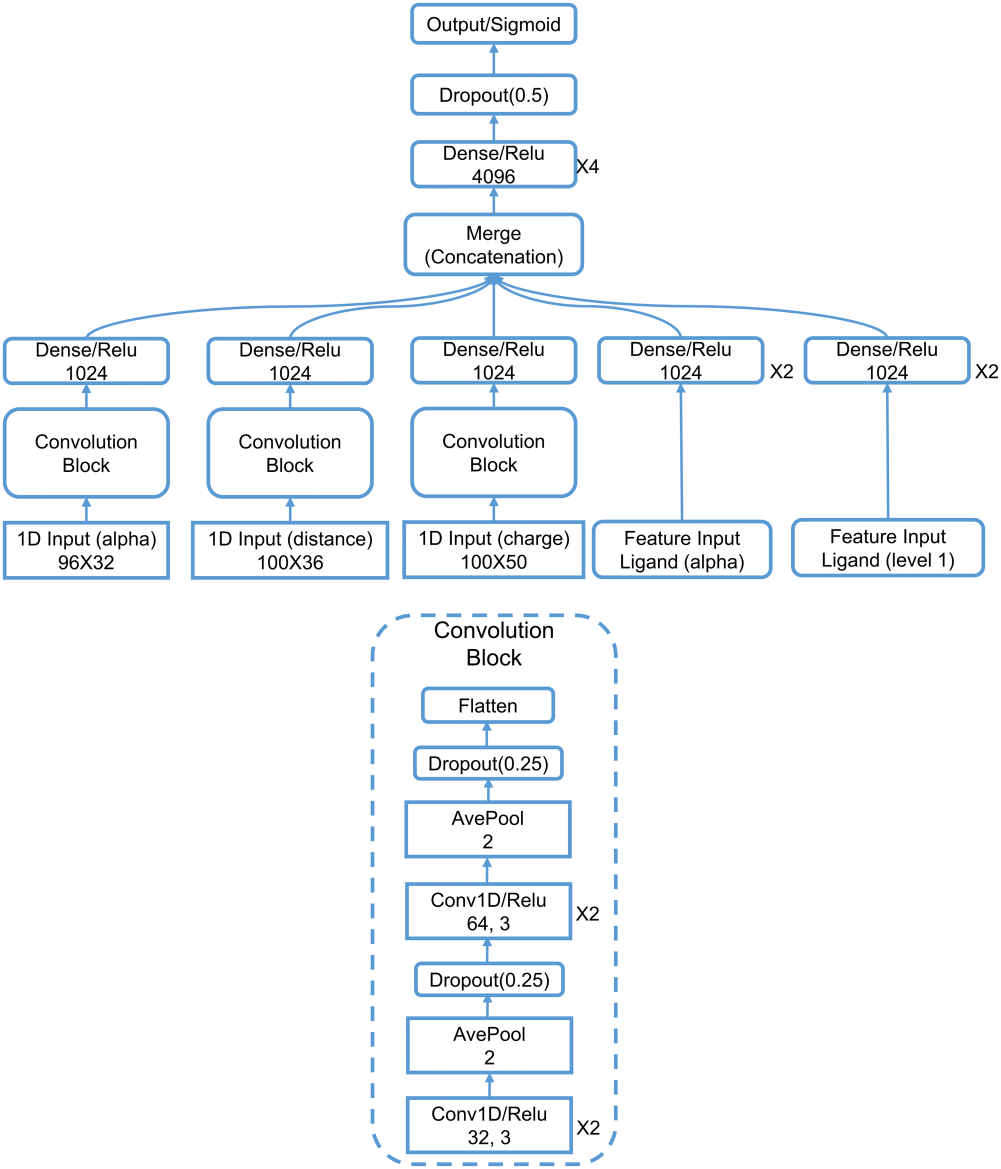
The network architecture of TopVS-DL. The 1D image-like layers are shown in sharp-corner rectangles. The numbers in convolution layers mean the number of filters and filter size from left to right. The pooling size of the pooling layers and dropout rate of the dropout layers are listed. The layers that are repeated *n* times are marked with “×*n*” sign on the right side of the layer.

The network architecture of TopVS-DL is shown in Fig 12. The Adam optimizer with learning rate set to 0.0001 is used. The loss function is binary cross-entropy. The network is trained with a batch size of 1024 and 10 epochs. The training data is shuffled for each epoch. The batch size is larger than that used in TopBP-DL due to the much larger training set in this problem. Because of the same reason, the training process converges to a small loss very fast with only a few training steps.

## Supporting information

S1 Text Extra results and records. Extra tables of detailed performance of PDBBind and DUD datasets, and the protein family exclusion in training set for the DUD dataset.
(PDF)

S1 Code Feature generation. Code for generating the features used in the final models in the Results section. It takes PDB files for proteins and Mol2 files for ligands as inputs.
(ZIP)
