# Supplementary material for: Representability of algebraic topology for biomolecules in machine learning based scoring and virtual screening
Source: PLoS Comput Biol. 2018 Jan 8;14(1):e1005929. doi: 10.1371/journal.pcbi.1005929 (PMC5774846; doi:10.1371/journal.pcbi.1005929)
Supplement: S1 Text — Extra tables of detailed performance of PDBBind and DUD datasets, and the protein family exclusion in training set for the DUD dataset. (PDF) [file pcbi.1005929.s001.pdf]

Supporting information
Representability of algebraic topology for biomolecules in machine learning based scoring and virtual screening

Zixuan Cang[1], Lin Mu[2], and Guo-Wei Wei[1,3,4] *
[1] Department of Mathematics
Michigan State University, MI 48824, USA
[2] Computer Science and Mathematics Division
Oak Ridge National Laboratory, Oak Ridge, USA
[3] Department of Biochemistry and Molecular Biology
Michigan State University, MI 48824, USA
[4] Department of Electrical and Computer Engineering
Michigan State University, MI 48824, USA

November 8, 2017

Feature	F^S Alpha complex							F^C Alpha complex						
Protein cluster	1	2	3	4	5	6	7	1	2	3	4	5	6	7
NOH	0.64(1.73)	0.79(1.30)	0.63(1.64)	**0.63(1.40)**	**0.82(1.36)**	0.63(1.68)	0.67(1.37)	0.53(1.96)	**0.76(1.39)**	0.45(2.05)	0.50(1.63)	**0.76(1.58)**	0.39(2.12)	0.57(1.58)
OSH	0.64(1.74)	0.79(1.30)	0.61(1.66)	**0.63(1.39)**	**0.79(1.45)**	0.56(1.79)	**0.78(1.17)**	0.45(2.10)	**0.75(1.41)**	0.37(2.16)	0.49(1.64)	0.74(1.63)	0.52(1.88)	0.64(1.47)
NOSH	0.65(1.72)	0.78(1.34)	0.64(1.61)	**0.64(1.38)**	**0.81(1.40)**	0.61(1.71)	0.67(1.37)	0.54(1.96)	0.74(1.44)	0.51(1.91)	**0.58(1.48)**	0.74(1.62)	0.40(2.09)	0.58(1.57)
CNS	0.62(1.77)	**0.82(1.23)**	0.65(1.61)	0.52(1.54)	**0.79(1.46)**	**0.65(1.65)**	0.70(1.32)	0.41(2.24)	0.72(1.53)	0.55(1.82)	0.50(1.70)	**0.75(1.64)**	**0.54(1.99)**	**0.69(1.38)**
CNO	0.62(1.77)	**0.82(1.23)**	**0.66(1.58)**	0.60(1.44)	0.78(1.48)	**0.64(1.65)**	0.60(1.49)	**0.58(1.90)**	**0.75(1.43)**	**0.58(1.79)**	**0.58(1.53)**	0.73(1.67)	0.51(1.99)	0.50(1.73)
OH	0.63(1.77)	0.79(1.30)	0.58(1.73)	0.60(1.44)	**0.80(1.43)**	0.56(1.79)	**0.78(1.16)**	0.51(1.98)	0.73(1.46)	0.43(2.03)	0.41(1.75)	**0.79(1.45)**	0.52(1.88)	**0.68(1.38)**
CN	0.63(1.77)	0.80(1.27)	0.64(1.63)	0.52(1.55)	0.78(1.48)	0.61(1.72)	**0.73(1.27)**	0.48(2.10)	0.74(1.47)	**0.58(1.78)**	0.41(1.83)	**0.76(1.59)**	0.51(2.07)	**0.71(1.35)**
CNOSPFClBrIH	0.64(1.74)	0.81(1.25)	**0.65(1.60)**	0.55(1.51)	0.73(1.63)	0.60(1.73)	**0.74(1.26)**	0.49(2.06)	0.71(1.55)	0.33(2.31)	0.35(1.94)	0.67(1.82)	**0.54(1.91)**	0.57(1.57)
COSH	**0.66(1.70)**	0.80(1.29)	**0.65(1.59)**	0.54(1.52)	0.74(1.61)	0.55(1.80)	0.72(1.28)	**0.60(1.83)**	0.69(1.58)	0.46(1.99)	**0.55(1.59)**	0.63(1.90)	0.48(2.02)	0.42(1.80)
CS	0.62(1.78)	**0.82(1.23)**	0.57(1.81)	0.59(1.45)	0.74(1.60)	**0.66(1.63)**	0.66(1.39)	0.50(2.09)	0.67(1.65)	0.52(1.90)	0.44(1.78)	0.72(1.71)	0.51(2.06)	0.60(1.50)
NH	0.64(1.74)	0.79(1.30)	0.61(1.66)	**0.63(1.39)**	0.78(1.48)	0.62(1.69)	0.57(1.53)	**0.62(1.79)**	0.72(1.50)	0.54(1.87)	**0.59(1.47)**	0.74(1.62)	0.48(1.94)	0.49(1.69)
CNOSH	0.64(1.75)	0.80(1.28)	**0.66(1.58)**	0.57(1.48)	0.73(1.62)	0.60(1.72)	0.64(1.42)	0.48(2.06)	0.73(1.50)	0.30(2.33)	0.33(1.94)	0.67(1.81)	**0.55(1.91)**	0.36(1.95)
NSH	0.63(1.75)	0.79(1.31)	0.63(1.63)	**0.65(1.37)**	0.76(1.55)	0.59(1.74)	0.57(1.53)	**0.63(1.78)**	0.70(1.54)	0.54(1.87)	**0.61(1.44)**	**0.75(1.59)**	**0.54(1.85)**	0.49(1.69)
CNOH	0.64(1.74)	0.80(1.28)	**0.65(1.59)**	0.56(1.49)	0.73(1.62)	0.59(1.74)	0.64(1.43)	0.53(1.98)	0.69(1.58)	0.53(1.87)	0.42(1.80)	0.66(1.83)	**0.55(1.89)**	0.42(1.83)
CNOS	0.58(1.85)	**0.82(1.23)**	0.62(1.66)	0.58(1.47)	0.78(1.50)	**0.65(1.65)**	0.59(1.51)	0.47(2.09)	0.73(1.50)	0.47(1.98)	0.47(1.72)	0.72(1.72)	0.52(1.97)	0.58(1.63)
CO	**0.65(1.72)**	**0.82(1.22)**	0.60(1.70)	0.55(1.50)	0.75(1.57)	0.59(1.76)	0.62(1.47)	0.58(1.89)	**0.74(1.44)**	**0.60(1.74)**	0.50(1.70)	0.65(1.89)	0.51(2.01)	0.58(1.58)
C	0.61(1.79)	0.80(1.27)	0.59(1.75)	0.52(1.55)	0.75(1.56)	0.64(1.68)	0.68(1.36)	0.54(2.00)	0.70(1.59)	0.51(1.89)	0.41(1.86)	0.70(1.76)	0.44(2.24)	0.55(1.59)
COH	**0.67(1.69)**	0.78(1.32)	0.61(1.65)	0.55(1.51)	0.71(1.67)	0.52(1.85)	0.73(1.28)	**0.58(1.87)**	0.70(1.55)	0.47(2.07)	0.39(1.81)	0.67(1.80)	0.44(2.08)	0.46(1.82)
CSH	0.65(1.72)	0.79(1.32)	0.56(1.74)	0.56(1.49)	0.71(1.68)	0.56(1.80)	**0.75(1.24)**	0.53(1.98)	0.70(1.54)	0.40(2.12)	0.53(1.62)	0.61(1.97)	0.43(2.12)	0.62(1.51)
CNOSPFClBrI	0.59(1.83)	0.81(1.24)	0.62(1.66)	0.56(1.50)	0.73(1.62)	**0.65(1.64)**	0.61(1.48)	0.44(2.15)	0.72(1.52)	0.50(1.94)	0.36(1.86)	0.64(1.91)	0.42(2.21)	0.53(1.71)
CNSH	0.64(1.74)	0.77(1.35)	0.61(1.66)	0.53(1.53)	0.72(1.65)	0.62(1.70)	0.66(1.40)	0.56(1.91)	0.73(1.47)	0.49(1.96)	0.49(1.68)	0.65(1.88)	0.43(2.15)	0.62(1.53)
H	0.63(1.76)	0.79(1.30)	0.58(1.71)	0.56(1.49)	0.78(1.49)	0.53(1.83)	0.66(1.39)	0.54(1.95)	**0.78(1.32)**	0.44(2.11)	0.53(1.56)	0.70(1.72)	0.40(2.05)	0.54(1.63)
COS	0.61(1.79)	0.82(1.23)	0.60(1.72)	0.57(1.48)	0.71(1.68)	0.63(1.68)	0.60(1.50)	0.52(2.00)	0.71(1.54)	0.54(1.85)	0.50(1.65)	0.63(1.98)	0.49(2.03)	0.54(1.64)
SH	0.62(1.78)	0.79(1.31)	0.58(1.74)	0.61(1.43)	0.74(1.60)	0.54(1.82)	0.65(1.41)	0.55(1.91)	0.73(1.46)	0.47(1.98)	0.50(1.62)	0.69(1.75)	0.46(1.95)	0.59(1.54)
CNH	**0.65(1.72)**	0.77(1.36)	0.60(1.69)	0.53(1.53)	0.71(1.67)	0.58(1.76)	0.66(1.39)	0.50(2.04)	0.70(1.56)	0.43(2.10)	0.51(1.62)	0.56(2.08)	0.35(2.33)	0.62(1.52)
CH	0.63(1.76)	0.77(1.36)	0.56(1.73)	0.54(1.52)	0.70(1.70)	0.52(1.86)	0.73(1.27)	0.46(2.11)	0.64(1.68)	0.41(2.07)	0.54(1.59)	0.62(1.91)	0.33(2.29)	**0.66(1.42)**
NO	0.65(1.72)	0.72(1.47)	0.62(1.65)	0.51(1.55)	0.59(1.95)	0.57(1.80)	0.64(1.44)	0.52(2.07)	0.62(1.75)	0.57(1.80)	0.50(1.61)	0.54(2.12)	0.38(2.28)	**0.64(1.49)**
NOS	**0.65(1.72)**	0.73(1.46)	0.50(1.93)	0.49(1.59)	0.56(2.01)	0.57(1.79)	0.65(1.44)	0.53(2.03)	0.60(1.78)	**0.57(1.79)**	0.48(1.66)	0.48(2.27)	0.34(2.36)	0.64(1.49)
NS	0.50(2.00)	0.40(1.97)	0.32(2.03)	0.41(1.67)	0.41(2.18)	0.44(1.98)	0.36(1.75)	0.50(1.99)	0.42(1.97)	0.37(1.95)	0.34(1.84)	0.23(2.45)	0.30(2.19)	0.41(1.70)
OS	0.54(1.94)	0.39(1.96)	0.52(1.84)	0.21(1.78)	0.04(2.41)	0.17(2.15)	0.60(1.52)	0.51(2.07)	0.37(2.00)	**0.57(1.75)**	0.18(1.83)	0.13(2.38)	0.15(2.21)	0.57(1.58)
N	0.45(2.04)	0.39(1.98)	-	0.38(1.69)	0.38(2.22)	0.42(2.01)	0.29(1.78)	0.41(2.09)	0.43(1.95)	-	0.39(1.73)	0.28(2.35)	0.33(2.12)	0.23(1.81)
O	0.55(1.92)	0.40(1.95)	0.37(1.96)	0.18(1.79)	0.00(2.43)	0.09(2.17)	0.59(1.55)	0.57(1.94)	0.35(2.02)	0.16(2.23)	0.12(1.82)	0.01(2.45)	0.12(2.16)	0.55(1.61)

Table A: Pearson correlation coefficients with RMSE (kcal/mol) in parentheses for predictions by different element type combinations for small-molecule characterization using alpha complex validated with 10-fold cross validation within each of the seven protein clusters in S1322. All experiments are repeated 20 times and the median Pearson correlation coefficient (RMSE in kcal/mol) are reported. The element combinations are ordered according to their average Pearson correlation coefficient in each row of the table. The top five best performing combinations for each protein cluster are highlighted in bold in each column.

*Address correspondences to Guo-Wei Wei. E-mail:wei@math.msu.edu

Protein cluster	1 (333)	2 (264)	3 (219)	4 (156)	5 (134)	6 (122)	7 (94)
CNOS	0.59/0.66/0.69/0.69	**0.81/0.84/0.82/0.84**	**0.57/0.68/0.60/0.69**	**0.66/0.68/0.65/0.71**	**0.72/0.79/0.66/0.79**	0.58/0.60/0.63/0.65	0.71/0.72/0.67/0.72
CNOSPFClBrI	0.60/0.65/0.69/0.68	**0.81/0.83/0.83/0.84**	**0.60/0.71/0.60/0.71**	**0.64/0.66/0.62/0.68**	**0.71/0.81/0.60/0.80**	0.60/0.59/0.64/0.66	0.72/0.69/0.65/0.70
NOSH	0.64/0.65/0.68/0.70	0.76/0.80/0.78/0.81	0.60/0.63/0.66/0.68	0.61/0.63/0.67/0.66	**0.79/0.82/0.73/0.84**	0.58/0.59/0.54/0.63	0.69/0.73/0.56/0.73
CNO	0.62/0.67/0.69/0.69	**0.81/0.83/0.81/0.84**	0.56/0.64/0.58/0.64	**0.67/0.68/0.61/0.70**	0.72/0.80/0.60/0.79	0.56/0.58/0.65/0.65	0.67/0.71/0.64/0.71
NOH	0.65/0.65/0.67/0.69	0.77/0.80/0.77/0.81	**0.62/0.65/0.59/0.68**	0.61/0.64/0.64/0.66	**0.80/0.83/0.71/0.84**	0.58/0.59/0.50/0.62	0.67/0.75/0.56/0.73
COS	0.62/0.66/0.67/0.69	0.80/0.82/0.81/0.83	0.53/0.64/0.59/0.67	**0.57/0.65/0.58/0.67**	0.65/0.74/0.63/0.75	0.55/0.59/0.62/0.64	**0.61/0.77/0.61/0.77**
CNOSPFClBrIH	0.64/0.67/0.66/0.68	**0.80/0.82/0.81/0.84**	0.60/0.67/0.60/0.65	0.65/0.61/0.59/0.60	0.68/0.76/0.68/0.78	**0.62/0.65/0.55/0.68**	**0.69/0.73/0.57/0.73**
CO	0.63/0.66/0.67/0.70	0.79/0.82/0.80/0.82	0.58/0.59/0.55/0.61	0.59/0.63/0.57/0.65	0.64/0.75/0.61/0.75	0.52/0.59/0.62/0.64	**0.59/0.73/0.60/0.76**
CNS	0.62/0.66/0.62/0.67	**0.79/0.82/0.83/0.84**	**0.56/0.63/0.65/0.68**	**0.64/0.66/0.65/0.68**	0.75/0.77/0.64/0.76	0.62/0.61/0.63/0.65	0.63/0.62/0.59/0.64
COH	**0.67/0.68/0.68/0.71**	0.78/0.81/0.75/0.81	0.62/0.67/0.57/0.67	0.51/0.55/0.54/0.57	0.67/0.76/0.63/0.77	**0.52/0.60/0.60/0.67**	**0.69/0.74/0.56/0.73**
CNOH	0.64/0.67/0.65/0.68	0.78/0.81/0.79/0.83	0.60/0.63/0.56/0.65	0.62/0.61/0.61/0.62	**0.71/0.77/0.67/0.80**	0.54/0.60/0.55/0.62	0.67/0.72/0.55/0.71
COSH	0.65/0.67/0.67/0.69	0.79/0.81/0.75/0.81	0.54/0.67/0.59/0.66	0.56/0.58/0.55/0.59	0.66/0.76/0.63/0.76	0.54/0.62/0.59/0.66	**0.69/0.74/0.58/0.73**
CNOSH	0.63/0.66/0.65/0.67	0.79/0.81/0.79/0.83	0.60/0.67/0.59/0.66	0.67/0.63/0.61/0.63	0.69/0.76/0.67/0.79	0.55/0.61/0.55/0.63	0.66/0.70/0.57/0.70
CNH	0.62/0.66/0.62/0.68	0.77/0.81/0.80/0.83	0.58/0.64/0.60/0.65	0.58/0.60/0.62/0.60	0.70/0.76/0.69/0.76	0.51/0.66/0.53/0.67	0.69/0.70/0.51/0.70
CNSH	0.65/0.66/0.62/0.67	0.77/0.81/0.81/0.83	0.52/0.61/0.60/0.63	0.59/0.60/0.62/0.61	0.70/0.76/0.70/0.76	0.53/0.65/0.55/0.67	0.68/0.70/0.51/0.69
CN	0.61/0.66/0.62/0.67	0.79/0.81/0.82/0.83	0.61/0.66/0.59/0.68	0.65/0.64/0.61/0.64	0.71/0.76/0.63/0.75	0.55/0.57/0.63/0.65	0.63/0.63/0.67/0.64
CCl	**0.66/0.70/0.63/0.71**	0.76/0.80/0.79/0.81	0.56/0.62/0.51/0.61	0.55/0.57/0.57/0.60	0.70/0.78/0.57/0.78	**0.54/0.61/0.64/0.68**	0.50/0.61/0.71/0.63
CSH	0.63/0.64/0.64/0.68	0.76/0.81/0.78/0.81	0.56/0.66/0.53/0.64	0.48/0.55/0.55/0.59	0.68/0.76/0.68/0.76	0.52/0.60/0.63/0.63	0.68/0.70/0.53/0.70
CClH	0.66/0.65/0.66/0.69	0.77/0.81/0.77/0.82	0.49/0.59/0.48/0.61	0.45/0.52/0.56/0.57	0.66/0.75/0.61/0.74	**0.52/0.61/0.64/0.67**	0.68/0.73/0.54/0.72
NSH	0.65/0.65/0.66/0.68	0.78/0.80/0.77/0.82	0.54/0.55/0.64/0.61	0.62/0.61/0.63/0.64	0.73/0.73/0.71/0.76	0.54/0.59/0.54/0.62	0.59/0.67/0.50/0.67
C	**0.65/0.70/0.63/0.71**	0.75/0.80/0.78/0.81	0.54/0.62/0.49/0.62	0.56/0.58/0.56/0.62	0.70/0.74/0.58/0.75	0.49/0.57/0.63/0.66	0.50/0.62/0.69/0.64
CS	0.64/0.68/0.63/0.69	0.76/0.80/0.79/0.82	0.54/0.60/0.55/0.62	0.55/0.61/0.54/0.62	0.69/0.74/0.60/0.74	**0.57/0.61/0.64/0.67**	0.51/0.62/0.68/0.64
CH	0.65/0.65/0.65/0.69	0.76/0.81/0.77/0.81	0.50/0.59/0.46/0.60	0.49/0.53/0.57/0.57	0.65/0.75/0.68/0.76	0.51/0.58/0.59/0.64	0.68/0.73/0.53/0.72
CBrH	0.65/0.65/0.65/0.69	0.76/0.80/0.77/0.81	0.48/0.59/0.46/0.60	0.49/0.54/0.57/0.58	0.66/0.75/0.67/0.77	0.51/0.59/0.60/0.64	0.68/0.72/0.54/0.71
CBr	**0.65/0.70/0.63/0.71**	0.76/0.80/0.78/0.81	0.52/0.61/0.50/0.61	0.56/0.61/0.59/0.65	0.70/0.75/0.58/0.74	0.49/0.57/0.64/0.66	0.51/0.61/0.58/0.60
OSH	0.61/0.63/0.68/0.69	0.76/0.76/0.71/0.78	**0.63/0.67/0.68/0.69**	0.50/0.54/0.62/0.62	0.73/0.74/0.63/0.76	0.50/0.48/0.55/0.54	0.73/0.72/0.55/0.71
OH	0.62/0.62/0.68/0.70	0.76/0.76/0.70/0.78	0.49/0.50/0.65/0.61	0.52/0.54/0.62/0.64	0.76/0.76/0.62/0.78	0.51/0.51/0.56/0.54	0.72/0.71/0.56/0.71
NH	0.65/0.65/0.67/0.68	0.80/0.81/0.77/0.82	0.58/0.58/0.58/0.62	0.57/0.60/0.58/0.60	0.75/0.75/0.71/0.78	0.56/0.60/0.50/0.59	0.59/0.66/0.50/0.67
SH	0.64/0.65/0.63/0.69	0.78/0.78/0.68/0.80	0.60/0.63/0.58/0.66	0.51/0.51/0.58/0.59	0.72/0.73/0.65/0.73	0.46/0.47/0.52/0.52	0.63/0.63/0.33/0.63
H	**0.64/0.64/0.67/0.71**	0.78/0.79/0.69/0.80	0.54/0.54/0.42/0.58	0.48/0.47/0.55/0.54	0.69/0.69/0.65/0.73	0.45/0.47/0.49/0.53	0.63/0.62/0.37/0.64
NO	0.57/0.56/0.56/0.63	0.65/0.64/0.61/0.64	0.55/0.57/0.58/0.62	0.49/0.48/0.41/0.50	0.56/0.59/0.62/0.66	0.52/0.52/0.48/0.57	0.66/0.64/0.58/0.65
NOS	0.54/0.58/0.54/0.62	0.63/0.64/0.62/0.65	0.55/0.60/0.57/0.59	0.38/0.47/0.43/0.51	0.56/0.60/0.65/0.66	0.52/0.54/0.48/0.61	0.65/0.64/0.55/0.63
NS	0.58/0.54/0.54/0.56	0.40/0.41/0.35/0.44	0.29/0.36/0.37/0.37	0.40/0.40/0.37/0.41	0.32/0.36/0.34/0.38	0.46/0.49/0.43/0.54	0.33/0.37/0.30/0.37
OS	0.52/0.56/0.56/0.62	0.43/0.43/0.37/0.42	0.55/0.53/0.54/0.59	0.13/0.08/0.05/0.16	0.00/0.02/-0.13/-0.04	0.23/0.20/0.17/0.20	0.56/0.57/0.06/0.55
N	0.46/0.45/0.44/0.47	0.40/0.40/0.37/0.43	0.04/-0.01/0.02/-0.01	0.32/0.31/0.36/0.39	0.28/0.34/0.32/0.37	0.38/0.38/0.48/0.46	0.28/0.27/0.08/0.24
O	0.54/0.53/0.55/0.62	0.43/0.43/0.40/0.43	0.17/0.16/0.30/0.42	-0.00/-0.02/0.12/-0.02	-0.19/-0.17/-0.14/-0.19	0.08/0.09/0.14/0.14	0.56/0.56/0.00/0.57
S	-0.16/-0.17/-0.16/-0.16	-0.20/-0.19/-0.19/-0.19	-0.20/-0.20/-0.19/-0.20	-0.23/-0.23/-0.24/-0.21	-0.29/-0.23/-0.25/-0.25	-0.27/-0.26/-0.23/-0.27	-0.29/-0.31/-0.28/-0.29
All	0.71/0.72/0.71/0.72	0.84/0.84/0.85/0.85	0.66/0.70/0.69/0.70	0.68/0.68/0.67/0.70	0.81/0.83/0.75/0.82	0.64/0.68/0.71/0.72	0.76/0.76/0.68/0.76

Table B: Pearson correlation coefficients for various predictions by F^S features for each element combination on 10-fold cross validation within each protein cluster in S1322 using Rips complex. Each experiment is repeated 20 times and the median results are reported. For each entry, from left to right are Pearson correlation coefficients for level 0 representation ($\mathbf{M}$ based persistent homology), level 0 plus level 1 enriched representation ($\widetilde{\mathbf{M}}^1$ based persistent homology), level 0 with electrostatics interaction based filtration, and level 0 plus level 1 with electrostatics interaction based filtration. In the majority of cases, level 1 enriched representation improves the performance, and distance based filtration and electrostatics interaction based filtration deliver comparable results.

Protein cluster	1	2	3	4	5	6	7
CNOS, 0, 0	0.60(1.84)	0.73(1.48)	0.45(1.96)	0.51(1.59)	0.61(1.94)	0.48(1.98)	0.60(1.52)
CNOS, 0, 1	0.46(2.14)	0.55(1.90)	0.36(2.09)	0.39(1.78)	0.36(2.39)	0.61(1.77)	0.56(1.56)
CNOS, 0, 2	0.39(2.22)	0.46(2.02)	0.20(2.34)	0.29(1.85)	0.10(2.62)	0.35(2.16)	0.56(1.57)
CNOSPFClBrI, 0, 0	0.55(1.95)	0.72(1.51)	0.44(1.97)	0.43(1.70)	0.58(2.04)	0.42(2.09)	0.54(1.65)
CNOSPFClBrI, 0, 1	0.47(2.09)	0.56(1.90)	0.38(2.06)	0.39(1.76)	0.35(2.40)	0.61(1.77)	0.56(1.56)
CNOSPFClBrI, 0, 2	0.39(2.22)	0.46(2.02)	0.20(2.34)	0.28(1.86)	0.10(2.62)	0.33(2.17)	0.56(1.57)
NOH, 0, 0	0.56(1.93)	0.67(1.61)	0.38(2.07)	0.61(1.45)	0.72(1.69)	0.20(2.33)	0.54(1.59)
NOH, 0, 1	0.49(2.06)	0.58(1.82)	0.29(2.19)	0.36(1.80)	0.45(2.29)	0.57(1.84)	0.58(1.51)
NOH, 0, 2	0.27(2.32)	0.16(2.21)	0.13(2.11)	0.14(2.14)	0.17(2.55)	0.04(2.26)	0.58(1.51)
CNO, 0, 0	0.59(1.90)	0.73(1.50)	0.55(1.80)	0.55(1.53)	0.60(1.98)	0.49(1.96)	0.59(1.53)
CNO, 0, 1	0.44(2.17)	0.52(1.89)	0.25(2.22)	0.37(1.76)	0.30(2.39)	0.60(1.79)	0.53(1.60)
CNO, 0, 2	0.39(2.22)	0.47(2.01)	0.06(2.34)	0.29(1.85)	0.06(2.66)	0.35(2.16)	0.56(1.57)
CNOSPFClBrIH, 0, 0	0.47(2.10)	0.70(1.55)	0.47(1.95)	0.49(1.63)	0.62(1.93)	0.33(2.18)	0.46(1.73)
CNOSPFClBrIH, 0, 1	0.48(2.05)	0.61(1.77)	0.32(2.14)	0.56(1.53)	0.50(2.13)	0.34(2.23)	0.58(1.53)
CNOSPFClBrIH, 0, 2	0.45(2.13)	0.46(2.06)	0.20(2.27)	0.22(2.00)	0.08(2.69)	0.26(2.27)	0.53(1.62)
CNOS, 1, 0	0.54(1.97)	0.74(1.45)	0.44(2.00)	0.47(1.66)	0.57(2.03)	0.51(1.94)	0.62(1.52)
CNOS, 1, 1	0.64(1.75)	0.73(1.47)	0.58(1.75)	0.49(1.63)	0.71(1.72)	0.47(2.01)	0.59(1.52)
CNOS, 1, 2	0.59(1.88)	0.74(1.46)	0.53(1.82)	0.46(1.66)	0.57(2.00)	0.45(2.02)	0.41(1.79)
CNOSPFClBrI, 1, 0	0.48(2.08)	0.74(1.47)	0.45(1.96)	0.40(1.72)	0.55(2.07)	0.48(1.98)	0.54(1.61)
CNOSPFClBrI, 1, 1	0.61(1.80)	0.72(1.50)	0.57(1.78)	0.52(1.58)	0.72(1.68)	0.51(1.93)	0.51(1.63)
CNOSPFClBrI, 1, 2	0.60(1.85)	0.73(1.48)	0.54(1.82)	0.55(1.53)	0.54(2.05)	0.51(1.93)	0.47(1.69)
NOH, 1, 0	0.43(2.17)	0.67(1.60)	0.40(2.05)	0.61(1.45)	0.68(1.79)	0.18(2.36)	0.45(1.71)
NOH, 1, 1	0.49(2.07)	0.58(1.81)	0.35(2.13)	0.37(1.76)	0.41(2.27)	0.47(2.01)	0.59(1.52)
NOH, 1, 2	0.51(2.07)	0.49(1.94)	0.08(2.31)	0.11(1.88)	0.41(2.58)	0.19(2.23)	0.58(1.55)
CNO, 1, 0	0.52(1.99)	0.72(1.50)	0.41(1.98)	0.53(1.57)	0.52(2.10)	0.44(2.05)	0.61(1.54)
CNO, 1, 1	0.61(1.80)	0.74(1.45)	0.56(1.79)	0.53(1.57)	0.73(1.67)	0.47(2.00)	0.60(1.51)
CNO, 1, 2	0.62(1.81)	0.75(1.44)	0.25(2.20)	0.44(1.69)	0.58(1.99)	0.45(2.05)	0.47(1.70)
CNOSPFClBrIH, 1, 0	0.43(2.18)	0.70(1.56)	0.44(1.96)	0.46(1.66)	0.63(1.89)	0.27(2.23)	0.41(1.79)
CNOSPFClBrIH, 1, 1	0.54(1.99)	0.70(1.54)	0.57(1.77)	0.36(1.80)	0.64(1.86)	0.56(1.87)	0.74(1.25)
CNOSPFClBrIH, 1, 2	0.61(1.84)	0.75(1.42)	0.49(1.90)	0.39(1.74)	0.50(2.11)	0.58(1.78)	0.59(1.52)
Averaged	0.70(1.66)	0.82(1.28)	0.62(1.68)	0.65(1.41)	0.76(1.68)	0.66(1.68)	0.74(1.31)

Table C: Pearson correlation coefficients with RMSE (kcal/mol) in parentheses for leave-one-out predictions by Wasserstein metric based K-nearest neighbor regression with different sets of barcodes for S1322. Rips complex filtration is applied. The entries in the first column are element combination, level of distance matrix (0 corresponds to $\mathbf{M}$ and 1 corresponds to $\widetilde{\mathbf{M}}^1$), and dimension of Betti numbers. The last row is the performance of the prediction by averaging over all the predictions with different induced distances.

	C	N	O	S	CN	CO	NO	CNO
C	0.49(2.63)	0.45(2.77)	0.41(2.80)	0.21(3.08)	0.52(2.56)	0.54(2.51)	0.41(2.74)	0.51(2.56)
N	0.31(2.97)	0.28(3.23)	0.28(3.11)	0.22(2.90)	0.29(3.00)	0.36(2.91)	0.35(2.94)	0.34(2.91)
O	0.22(3.06)	0.21(3.16)	0.24(3.11)	0.01(3.07)	0.27(3.00)	0.20(3.09)	0.22(3.12)	0.27(2.98)
S	0.19(2.95)	0.20(3.01)	0.16(3.07)	0.23(2.89)	0.15(3.00)	0.12(3.03)	0.10(3.10)	0.10(3.12)
P	0.09(2.96)	0.12(2.94)	0.07(2.99)	-0.02(2.98)	0.10(2.96)	0.07(2.98)	0.03(2.98)	0.06(2.98)
F	0.11(2.97)	0.09(3.00)	0.07(3.02)	0.09(2.97)	0.08(2.99)	0.15(2.94)	0.17(2.92)	0.18(2.92)
Cl	0.15(2.94)	0.02(3.05)	0.03(3.05)	0.09(2.97)	0.17(2.93)	0.10(2.98)	0.04(3.03)	0.09(2.99)
Br	0.06(2.96)	0.07(2.96)	0.01(2.99)	0.07(2.96)	0.06(2.97)	0.01(2.99)	-0.01(3.03)	0.07(2.96)
I	0.08(2.96)	0.01(2.98)	0.07(2.96)	0.11(2.95)	0.02(2.97)	0.09(2.95)	0.09(2.95)	0.07(2.96)
CN	0.48(2.62)	0.47(2.72)	0.45(2.71)	0.31(2.92)	0.53(2.53)	**0.59(2.40)**	0.51(2.58)	0.49(2.59)
CO	0.54(2.52)	0.49(2.68)	0.54(2.54)	0.27(2.96)	**0.59(2.40)**	**0.60(2.39)**	0.55(2.52)	0.51(2.59)
CS	0.51(2.56)	0.44(2.80)	0.43(2.76)	0.23(3.11)	0.54(2.53)	**0.56(2.47)**	0.38(2.83)	0.50(2.59)
NO	0.38(2.87)	0.34(3.02)	0.28(3.08)	0.15(3.07)	0.39(2.84)	0.30(2.98)	0.30(2.96)	0.30(2.97)
NS	0.35(2.90)	0.26(3.24)	0.28(3.08)	0.09(3.01)	0.34(2.95)	0.41(2.80)	0.40(2.83)	0.40(2.79)
OS	0.17(3.16)	0.20(3.19)	0.27(3.17)	-0.06(3.15)	0.23(3.09)	0.17(3.14)	0.15(3.21)	0.29(2.94)
CNO	**0.56(2.51)**	0.51(2.60)	0.48(2.67)	0.33(2.91)	**0.61(2.37)**	0.56(2.49)	0.55(2.51)	**0.61(2.36)**
CNS	0.51(2.57)	0.45(2.76)	0.44(2.73)	0.31(2.90)	0.49(2.60)	**0.59(2.40)**	0.48(2.62)	0.53(2.53)
COS	**0.56(2.47)**	0.52(2.61)	**0.57(2.47)**	0.27(2.97)	**0.57(2.45)**	**0.61(2.37)**	0.55(2.51)	0.54(2.53)
NOS	0.43(2.77)	0.37(2.95)	0.32(3.04)	0.25(2.90)	0.40(2.79)	0.31(2.95)	0.32(2.94)	0.35(2.88)
CNOS	**0.58(2.44)**	0.51(2.64)	0.49(2.65)	0.31(2.93)	**0.62(2.34)**	0.54(2.53)	**0.56(2.47)**	**0.59(2.42)**

Table D: Pearson correlation coefficients with RMSE (kcal/mol) in parentheses for predictions of the PDBBind v2016 core set by different combinations of element types for protein (columns) and ligand (rows) using Rips complex with interactive persistent homology and F^C features based on the bins, $\{[0, 2.5), [2.5, 3), [3, 3.5), [3.5, 4.5), [4.5, 6), [6, 12]\}$. The models are trained with the PDBBind v2016 refined set, excluding the PDBBind v2016 core set. Only Betti-0 information is collected. The top 16 combinations are marked in bold.

	C	N	O	CN	CO	NO	CNO	CNOS
C	0.76(1.98)	**0.79(1.96)**	0.77(2.01)	0.76(2.02)	0.75(2.02)	**0.78(1.95)**	0.76(2.01)	0.76(2.00)
N	0.75(2.02)	0.74(2.05)	0.75(2.07)	0.75(2.02)	0.75(2.02)	0.74(2.06)	0.75(2.05)	0.76(2.02)
O	0.75(2.02)	0.72(2.12)	0.74(2.07)	0.75(2.02)	0.73(2.09)	0.74(2.06)	0.73(2.08)	0.74(2.06)
S	0.70(2.15)	0.70(2.17)	0.71(2.16)	0.72(2.12)	0.70(2.16)	0.72(2.10)	0.71(2.13)	0.73(2.09)
CN	**0.78(1.94)**	**0.78(1.96)**	**0.78(1.98)**	0.76(1.99)	0.76(2.00)	**0.78(1.94)**	0.77(1.97)	**0.78(1.96)**
CO	0.76(2.00)	0.77(1.98)	0.75(2.05)	0.74(2.06)	0.75(2.05)	0.76(2.00)	0.75(2.04)	0.75(2.04)
CS	0.77(1.96)	**0.79(1.95)**	0.77(2.00)	0.76(2.01)	0.76(1.99)	**0.78(1.95)**	0.77(1.99)	0.77(1.98)
NO	0.73(2.09)	0.74(2.08)	0.71(2.16)	0.73(2.10)	0.72(2.13)	0.73(2.11)	0.72(2.11)	0.73(2.10)
NS	0.72(2.09)	0.72(2.12)	0.72(2.13)	0.71(2.12)	0.70(2.14)	0.72(2.11)	0.70(2.16)	0.71(2.13)
OS	0.74(2.03)	0.73(2.09)	0.75(2.04)	0.74(2.03)	0.72(2.10)	0.74(2.05)	0.73(2.07)	0.74(2.04)
CNO	0.75(2.03)	0.77(1.99)	0.76(2.05)	0.74(2.06)	0.73(2.08)	0.75(2.03)	0.75(2.04)	0.76(2.04)
CNS	**0.78(1.93)**	**0.78(1.95)**	**0.78(1.98)**	0.76(1.99)	0.76(1.99)	**0.78(1.94)**	**0.77(1.96)**	**0.78(1.95)**
COS	0.76(1.99)	**0.78(1.97)**	0.76(2.04)	0.75(2.04)	0.75(2.04)	0.76(1.99)	0.76(2.03)	0.76(2.02)
NOS	0.74(2.06)	0.74(2.07)	0.71(2.17)	0.73(2.09)	0.71(2.13)	0.73(2.10)	0.72(2.12)	0.73(2.10)
CNOS	0.75(2.03)	0.77(2.00)	0.75(2.06)	0.74(2.05)	0.73(2.08)	0.75(2.03)	0.75(2.03)	0.76(2.02)
CNOSPFClBrI	0.75(2.03)	0.77(1.99)	0.75(2.08)	0.74(2.05)	0.75(2.06)	0.75(2.04)	0.76(2.03)	0.76(2.02)

Table E: Pearson correlation coefficients with RMSE (kcal/mol) in parentheses for predictions of the PDBBind v2016 core set by different combinations of element types for protein (columns) and ligand (rows) using alpha complex with F^S features. The models are trained with the PDBBind v2016 refined set, excluding the PDBBind v2016 core set. Betti-0, Betti-1, and Betti-2 barcodes are considered. The top 16 combinations are marked in bold.

Target	Proteins excluded from training set of the target
ACE	ACE, ADA, COMT, PDE5, ALR2,
AChE	AChE, ALR2, AmpC, COX1, COX2, GPB, HIVPR, HIVRT, HMGR, InhA, NA, PARP, PNP, SAHH, FXa,
ADA	ACE, ADA, COMT, PDE5,
ALR2	AChE, ALR2, AmpC, COX1, COX2, GPB, HIVPR, HIVRT, HMGR, InhA, NA, PARP, PNP, SAHH, GART, ACE, RXRa, PPARg, AmpC, COX1, COX2,
AmpC	AChE, ALR2, AmpC, COX1, COX2, GPB, HIVPR, HIVRT, HMGR, InhA, NA, PARP, PNP, SAHH, GART, ACE, RXRa, PPARg, ALR2, COX1, COX2,
AR	AR, TK, ADA, ALR2, PARP, PNP, SAHH, ER_agonist, ER_antagonist, GR, MR, PPARg, PR, RXRa,
CDK2	CDK2, EGFr, FGFr1, HSP90, P38 MAP, PDGFrb, SRC, TK, VEGFr2,
COMT	ACE, ADA, COMT, PDE5, RXRa, ALR2, AmpC, PNP,
COX1	AChE, ALR2, AmpC, COX1, COX2, GPB, HIVPR, HIVRT, HMGR, InhA, NA, PARP, PNP, SAHH, ALR2, COX2,
COX2	AChE, ALR2, AmpC, COX1, COX2, GPB, HIVPR, HIVRT, HMGR, InhA, NA, PARP, PNP, SAHH, HSP90, ALR2, PARP,
DHFR	GART, DHFR, PPARg,
EGFr	CDK2, EGFr, FGFr1, HSP90, P38 MAP, PDGFrb, SRC, TK, VEGFr2,
ER_agonist	ER_agonist, PNP, AR, ER_antagonist, GR, MR, PPARg, PR, RXRa,
ER_antagonist	AR, ER_agonist, ER_antagonist, GR, MR, PPARg, PR, RXRa,
FGFr1	CDK2, EGFr, FGFr1, HSP90, P38 MAP, PDGFrb, SRC, TK, VEGFr2,
FXa	FXa, thrombin, trypsin, DHFR, GART,
GART	GART, DHFR, PPARg,
GPB	AChE, ALR2, AmpC, COX1, COX2, GPB, HIVPR, HIVRT, HMGR, InhA, NA, PARP, PNP, SAHH, COMT,
GR	AR, ER_agonist, ER_antagonist, GR, MR, PPARg, PR, RXRa,
HIVPR	AChE, ALR2, AmpC, COX1, COX2, GPB, HIVPR, HIVRT, HMGR, InhA, NA, PARP, PNP, SAHH,
HIVRT	AChE, ALR2, AmpC, COX1, COX2, GPB, HIVPR, HIVRT, HMGR, InhA, NA, PARP, PNP, SAHH, PNP,
HMGR	AChE, ALR2, AmpC, COX1, COX2, GPB, HIVPR, HIVRT, HMGR, InhA, NA, PARP, PNP, SAHH, RXRa, ACE, GART, ALR2, AmpC, COX1,
HSP90	CDK2, EGFr, FGFr1, HSP90, P38 MAP, PDGFrb, SRC, TK, VEGFr2,
InhA	AChE, ALR2, AmpC, COX1, COX2, GPB, HIVPR, HIVRT, HMGR, InhA, NA, PARP, PNP, SAHH,
MR	AR, ER_agonist, ER_antagonist, GR, MR, PPARg, PR, RXRa, PARP,
NA	AChE, ALR2, AmpC, COX1, COX2, GPB, HIVPR, HIVRT, HMGR, InhA, NA, PARP, PNP, SAHH, PPARg, thrombin, trypsin, ADA,
P38 MAP	CDK2, EGFr, FGFr1, HSP90, P38 MAP, PDGFrb, SRC, TK, VEGFr2,
PARP	AChE, ALR2, AmpC, COX1, COX2, GPB, HIVPR, HIVRT, HMGR, InhA, NA, PARP, PNP, SAHH, COX1, PNP,
PDE5	ACE, ADA, COMT, PDE5, P38 MAP,
PDGFrb	CDK2, EGFr, FGFr1, HSP90, P38 MAP, PDGFrb, SRC, TK, VEGFr2,
PNP	AChE, ALR2, AmpC, COX1, COX2, GPB, HIVPR, HIVRT, HMGR, InhA, NA, PARP, PNP, SAHH, TK, ADA, COMT, COX1, GPB, PARP, SAHH,
PPARg	AR, ER_agonist, ER_antagonist, GR, MR, PPARg, PR, RXRa,
PR	AR, ER_agonist, ER_antagonist, GR, MR, PPARg, PR, RXRa,
RXRa	AR, ER_agonist, ER_antagonist, GR, MR, PPARg, PR, RXRa, COX1,
SAHH	AChE, ALR2, AmpC, COX1, COX2, GPB, HIVPR, HIVRT, HMGR, InhA, NA, PARP, PNP, SAHH, TK, ADA, COMT, COX1, PARP, GPB, PNP,
SRC	CDK2, EGFr, FGFr1, HSP90, P38 MAP, PDGFrb, SRC, TK, VEGFr2, PDE5,
thrombin	FXa, thrombin, trypsin, DHFR, ER_antagonist,
TK	CDK2, EGFr, FGFr1, HSP90, P38 MAP, PDGFrb, SRC, TK, VEGFr2, ADA, COMT, ALR2, COX1, GPB, PARP, PNP, SAHH,
trypsin	FXa, thrombin, trypsin, PPARg, ADA, DHFR,
VEGFr2	CDK2, EGFr, FGFr1, HSP90, P38 MAP, PDGFrb, SRC, TK, VEGFr2,

Table F: List of proteins that are excluded from the training set of each target in the DUD dataset.

Run #	$EF_{2\%}$	$EF_{20\%}$	AUC
1	8.63	3.42	0.830
2	8.57	3.47	0.832
3	8.61	3.43	0.830
4	8.52	3.42	0.831
5	8.36	3.47	0.834
6	8.39	3.45	0.833
7	8.59	3.43	0.831
8	8.94	3.47	0.832
9	8.77	3.42	0.832
10	8.62	3.46	0.832
Median	8.60	3.45	0.832
Std	0.16	0.02	1.2E-3

Table G: The DUD test results of TopVS-ML for each repeated run. Median and Std are the median value and the standard deviation across repeated runs.

Target	ADV	LIG	COM	ALL
AR	0.81	0.83	0.93	0.90
COX2	0.86	0.97	0.80	0.97
DHFR	0.82	0.95	0.94	0.96
$ER_{agonist}$	0.84	0.69	0.91	0.81
MR	0.82	0.78	0.91	0.89
PPARg	0.82	0.70	0.72	0.72
RXRa	0.95	0.74	0.91	0.79
SAHH	0.80	0.81	0.72	0.84
Average	0.84	0.81	0.86	0.86

Table H: The AUC for autodock vina, TopVS-ML with only compound features, TopVS-ML with only protein-compound complex features, and TopVS-ML with all features. The targets with high quality results by Autodock Vina are reported (AUC > 0.8)

Target	ADV	LIG	COM	ALL
ACE	0.42	0.85	0.78	0.81
ADA	0.49	0.89	0.89	0.89
AmpC	0.34	0.56	0.37	0.53
FGFr1	0.44	0.97	0.71	0.95
GPB	0.48	0.70	0.69	0.71
NA	0.37	0.79	0.82	0.84
PDGFrb	0.32	0.98	0.90	0.96
Average	0.41	0.82	0.74	0.81

Table I: The AUC for autodock vina, TopVS-ML with only compound features, TopVS-ML with only protein-compound complex features, and TopVS-ML with all features. The targets with low quality results by Autodock Vina are reported (AUC < 0.5)
